# Socioeconomic differences in the utilization of diagnostic imaging and non-pharmaceutical conservative therapies for spinal diseases

**DOI:** 10.1186/s12891-023-06909-6

**Published:** 2023-10-02

**Authors:** Falko Tesch, Jochen Schmitt, Patrik Dröge, Christian Günster, Andreas Seidler, Johannes Flechtenmacher, Burkhard Lembeck, Bernd Kladny, Dieter Christian Wirtz, Fritz-Uwe Niethard, Toni Lange

**Affiliations:** 1 Center for Evidence-Based Healthcare, University Hospital and Faculty of Medicine Carl Gustav Carus, TU Dresden, 01307 Dresden, Germany; 2 Allgemeine Ortskrankenkasse (AOK) Research Institute, Berlin, Germany; 3 Institute and Policlinic of Occupational and Social Medicine (IPAS), Faculty of Medicine Carl Gustav Carus, TU Dresden, Dresden, Germany; 4 German Professional Association for Orthopedics and Trauma Surgery, Berlin, Germany; 5 Department of Orthopedics and Trauma Surgery, m&i Fachklinik Herzogenaurach, Herzogenaurach, Germany; 6 Department of Orthopedics and Trauma Surgery, University Hospital Bonn, Bonn, Germany

**Keywords:** Spinal diseases, Back pain, Routine healthcare data, Healthcare disparities

## Abstract

**Background:**

A different utilization of health care services due to socioeconomic status on the same health plan contradicts the principle of equal treatment. We investigated the presence and magnitude of socioeconomic differences in utilization of diagnostic imaging and non-pharmaceutical conservative therapies for patients with spinal diseases.

**Methods:**

The cohort study based on routine healthcare data from Germany with 11.7 million patient-years between 2012 and 2016 for patients with physician-confirmed spinal diseases (ICD-10: M40-M54), occupation and age 20 to 64 years. A Poisson model estimated the effects of the socioeconomic status (school education, professional education and occupational position) for the risk ratio of receiving diagnostic imaging (radiography, computed tomography, magnetic resonance imaging) and non-pharmaceutical conservative therapies (physical therapy including exercise therapy, manual therapy and massage, spinal manipulative therapy, acupuncture).

**Results:**

Patients received diagnostic imaging in 26%, physical therapy in 32%, spinal manipulative therapy in 25%, and acupuncture in 4% of all patient-years. Similar to previous survey-based studies higher rates of utilization were associated with higher socioeconomic status. These differences were most pronounced for manual therapy, exercise therapy, and magnetic resonance imaging.

**Conclusions:**

The observed differences in health care utilization were highly related to socioeconomic status. Socioeconomic differences were higher for more expensive health services. Further research is necessary to identify barriers to equitable access to health services and to take appropriate action to decrease existing social disparities.

**Supplementary Information:**

The online version contains supplementary material available at 10.1186/s12891-023-06909-6.

## Background

A different utilization of health services by different groups regarding their socioeconomic status (SES) on the same health plan contradicts the principle of equal treatment. This principle could also apply to a country like Germany, which has a universal health care system with free choice of physicians and compulsory health insurance. The costs of health care are covered for the majority of the population and benefits by income-related contributions from the insured. According to the “theory of fundamental causes” by BG Link and J Phelan [1], individual risk factors are always embedded in a social context that makes people more or less susceptible to disease. This implies that regardless of the level of medical care, disadvantaged groups are more likely to develop a disease. In contrast, groups with high socioeconomic status (SES) use more resources (e.g., knowledge, money, prestige, power, contacts) to protect themselves from disease [1].

The SES can be approximated through different variables. The most common ones are household income, occupational position and education level. Differences in mortality associated to occupational status, employment status, education and income are known for Germany as well as other countries [2, 3]. This implied also SES differences in health and healthcare. Higher educational and occupational standing were for instance found to be positively associated with the utilization of outpatient specialists, while no consistent differences across different countries were found for the size of the social networks or for those with financial strains [4]. Furthermore, higher educational and occupational standing might also increase the risk ratio for diagnostic tests [5].

This issue is particularly important for common diseases with a high burden such as spinal diseases [6]. Regarding the impact of the SES on the utilization of health care services for spinal diseases, research on diagnostic imaging has been based on the estimation of income for the SES from routine healthcare data [7–9], while evidence on the impact of SES on non-pharmaceutical conservative therapies is derived from survey data only [10–15]. Overall, based on different estimations of the SES, studies indicated an impact of the SES on the utilization of diagnostic imaging and non-pharmaceutical conservative therapies. However, comparability and transferability of study results is hindered by the use of different estimates and different types of data sources. To the best of our knowledge, there is currently a lack of studies investigating the impact of SES on the utilization of health services for spinal diseases. Therefore, the objective of this study was to estimate the magnitude of the socioeconomic differences in utilization of diagnostic imaging and non-pharmaceutical conservative therapies for spinal diseases in Germany.

## Methods

### Data

We undertook a cohort study based on comprehensive routine healthcare data from Germany for the period 2012 to 2016. In Germany, about 90% of the population are members of a statutory health insurance program. We used routine healthcare data collected by the “Allgemeine Ortskrankenkassen” (AOK), which is the largest statutory health insurance in Germany representing about 24 million people.

### Inclusion and exclusion criteria

The study cohort consisted of insured persons with a prevalent, physician-confirmed spinal disease according to the 10th revision of the International Statistical Classification of Diseases and Related Health Problems (ICD-10) diagnoses M40-M54 (deforming dorsopathies, spondylopathies, intervertebral disc disorders and dorsalgia) in the outpatient or inpatient sector. Of these, those with an occupation, with an age between 20 and 64 years and a residence in Germany were selected. Patients were excluded if they had a concomitant fracture of the spine (ICD-10: S12, S22, S32) or less than 350 insurance days per year (patients who died during the year were still included).

### Outcomes

Primary study outcomes were the presents of diagnostic imaging and non-pharmaceutical conservative therapies as binary variables. Diagnostic imaging was further sub-classified into radiography, computed tomography and magnetic resonance imaging. The non-pharmaceutical conservative therapies were further divided into spinal manipulative therapy, acupuncture (indication for the spine or knee) done by the physician and physical therapy done by a physiotherapist. The latter was further divided into the primary therapies exercise therapy, manual therapy and massage. Unlike for medications, we were able to verify that the diagnostic/therapy was indicated for the spine.

### Exposure

In Germany notifications to statutory social insurances about the job role are mandatory. Changes of the German job role code in 2011 made it also possibly to approximate the SES much better than before. The 9-digit “German job role code” provides information about the “occupational field” (1-4th digit), the “occupational position” of the employee (5th digit), “school education” (6th digit) and “professional education” (7th digit) and “part-time/temporary work” (8th /9th digit) . It was possible to report unknown school and/or unknown professional education by the employer for the employee. Information for “school education”, “professional education” and “occupational position” were used as variables to estimate the SES.

Within “professional education” the “German job role code” distinguishes between those with vocational training, those with additional qualifications beyond their vocational training such as master craftsman or a technician degree and those with various academic qualifications. Due to the late introduction of the Bachelor/Master system in Germany all college degrees where combined in one category. Overall, 1286 different occupations are coded in the job role code.

Furthermore, all of them are divided into four different occupational positions such as “Helper”, “Trained“, „Specialist” and “Expert”. The position “Helper” describes simple work with highly structured working processes. The position “Trained” describes a specialized work, which often required a vocational training in the specific field. The “Specialist” position describes complex work, which often required a college degree, but with limited decision-making authority. The “Expert” position represents occupations with a high degree of job control, which often also includes the management of people [16]. For a better understanding, we refer to the highest occupational position as “Management”.

### Control variables

The following variables were used for adjustment: sex, age, sick day leaves, amount of quarters with spine diagnoses, certain comorbidities, pain medication and consulted physicians (general practitioner, orthopedic practitioner, surgeon, and preventive and rehabilitation physician). The corresponding definitions with respective codes in their classifications system can be accessed through Supplementary table S1-S3. Furthermore, the models were adjusted for calendar year, the 96 German spatial planning regions and the 10 occupational fields of the 9-digit “German job role code”. Base models adjusted for region, calendar year and each variable can be found in Table S6 and for all used variables in Table S5.

### Statistical analysis

The outcomes diagnostic imaging and non-pharmaceutical conservative therapies in each year were modeled as binary variables with a generalized linear model with a Poisson error distribution and a log link function to estimate multivariable-adjusted relative risks (RR) with 95% confidence intervals (95% CI). Poisson model yields consistent estimators of model coefficients irrespective of the distribution of the outcome [17]. The analysis was done via remote data processing. Due to the size of the data, the statistical modeling was performed using the R package “speedglm” version 0.3-2 in the statistical software R [18]. Because of the adjustment for rheumatic diseases with spinal involvement (1.2% of all patient years), the calculated effects apply to all patients with spinal diseases except these. Forest plots were used to show the results for each outcome graphically. The reference category for the SES variables was set to the highest school educational level (High school diploma), the highest professional education (College) and to the lowest occupational category (Helper).

## Results

### Cohort description

Overall, the average number of patients per year was 2.9 million, resulting in 11.6 million patient-years with spinal diseases observed in 2012–2016. Both sexes were equally distributed. In 75% of all patient-years a consultation with a general practitioner for the ICD-10 diagnosis M40-M54 was documented. An orthopedic practitioner was consulted in 31.5%, a surgeon was consulted in 5.3% and a physician with the specialty physical therapy and rehabilitation medicine (PRM) was consulted in 2.3% of all patient-years. In about half of all patient-years non-steroidal anti-inflammatory drugs (NSAID) pain medication like acetylsalicylic acid, Ibuprofen and Diclofenac were prescribed. The second most common medication group were non-opioid analgesics like Metamizol and Paracetamol being prescribed in 18.9% of all patient-years. As comorbid musculoskeletal diseases Osteoarthritis of the hip (6.8%), the knee (3.5%) as well as Osteoporosis (1.6%) were present.

For those with known education most patient-years were reported as either low secondary school leaving certificate or intermediate school leaving certificate after nine/ten years of education. 55.3% had vocational training as their highest professional grade. This corresponds with the most common occupational position “Trained” (60.2%), compared to 28.3% of all patients years with a “Helper” position (Table 1).

**Table 1:** Baseline characteristic of the study population for years with spinal diseases, spinal imaging and physical therapy for the spine in 2012 to 2016 in AOK

	Spinal Disease	
	Patient years with	%
**Total**	11,692,528	100
Sociodemographic*		
Male	5,830,821	49.9
Female	5,861,707	50.1
Age group 20–34	2,397,952	20.5
Age group 35–49	4,142,133	35.4
Age group 50–64	5,152,443	44.1
***Comorbidities***		
Osteoarthritis (knee)	403,515	3.5
Osteoarthritis (hip)	790,966	6.8
Osteoporosis	181,882	1.6
Chronic polyarthritis	185,862	1.6
Rheumatic diseases (With typical spine involvement)	137,829	1.2
Rheumatic diseases (Without typical spine involvement)	83,690	0.7
Depression	1,696,999	14.5
Anxiety disorder	580,462	5.0
Psychosomatic disorders	1,157,579	9.9
Sleep disorders	649,749	5.6
**Physician consultations with diagnosis spinal diseases**		
General practitioner	8,769,549	75.0
Orthopedic practitioner	3,683,485	31.5
Surgeon	622,372	5.3
physical therapy and rehabilitation physician	264,136	2.3
Pain medication*		
Nonsteroidal anti-inflammatory drugs	5,934,967	50.8
Cox-2 inhibitors	415,060	3.5
Non-opioid analgesics	2,214,495	18.9
Weak opioids	600,652	5.1
Strong opioids	79,864	0.7
Sick days leave*		
None	7,322,939	62.6
1 and more days	4,369,589	37.4
**School education**		
Unknown school-leaving qualification	3,987,669	34.1
No school-leaving qualification	307,915	2.6
Lower Secondary leaving certificate	3,477,109	29.7
Intermediate school leaving certificate	2,894,126	24.8
High school diploma	1,025,708	8.8
**Professional education**		
Unknown vocational training	2,453,842	21.0
Without vocational training	2,021,184	17.3
With vocational training	6,464,084	55.3
Master craftsman/technician degree	353,674	3.0
College degree	399,743	3.4
**Occupation**		
Agriculture	256,368	2.2
Raw material extraction, production	3,107,581	26.6
Construction	864,378	7.4
Natural science	214,548	1.8
Transport, logistics and security	2,621,758	22.4
Commercial services, distribution, tourism	1,332,634	11.4
Business and law	1,335,204	11.4
Health, social services, teaching and education	1,797,835	15.4
Language, media, art, culture and design	161,278	1.4
Military	944	0.0
Position „Helper“	3,313,943	28.3
Position „Trained“	7,042,991	60.2
Position „Specialist“	857,946	7.3
Position „Management“	477,647	4.1

* Age groups, sick days leave and pain medication have been aggregated and years, quarters with diagnosis and regional distribution of the patients is not shown

### Regression results for diagnostic imaging

About 3.1 million patient-years experienced a diagnostic imaging of the spine, which accounted for 26.3% of all patient-years. The most common diagnostic imaging were radiography with 18.6%, magnetic resonance imaging with 10.6% and computed tomography with 2.2% of the patient-years (Supplementary table S4). The patient-years with diagnostic imaging had more comorbidities, sick days leave, higher pain medication usage and also had more consultation by specialist physicians than those with only a diagnosis of a spinal disease. The results of the regression models show that with increasing age the risk ratio of computed tomography increases, while the risk ratio of radiography decreases. A consultation with an orthopedic practitioner was associated with more spinal imaging, especially radiography (Supplementary table S5).

**Fig. 1:**
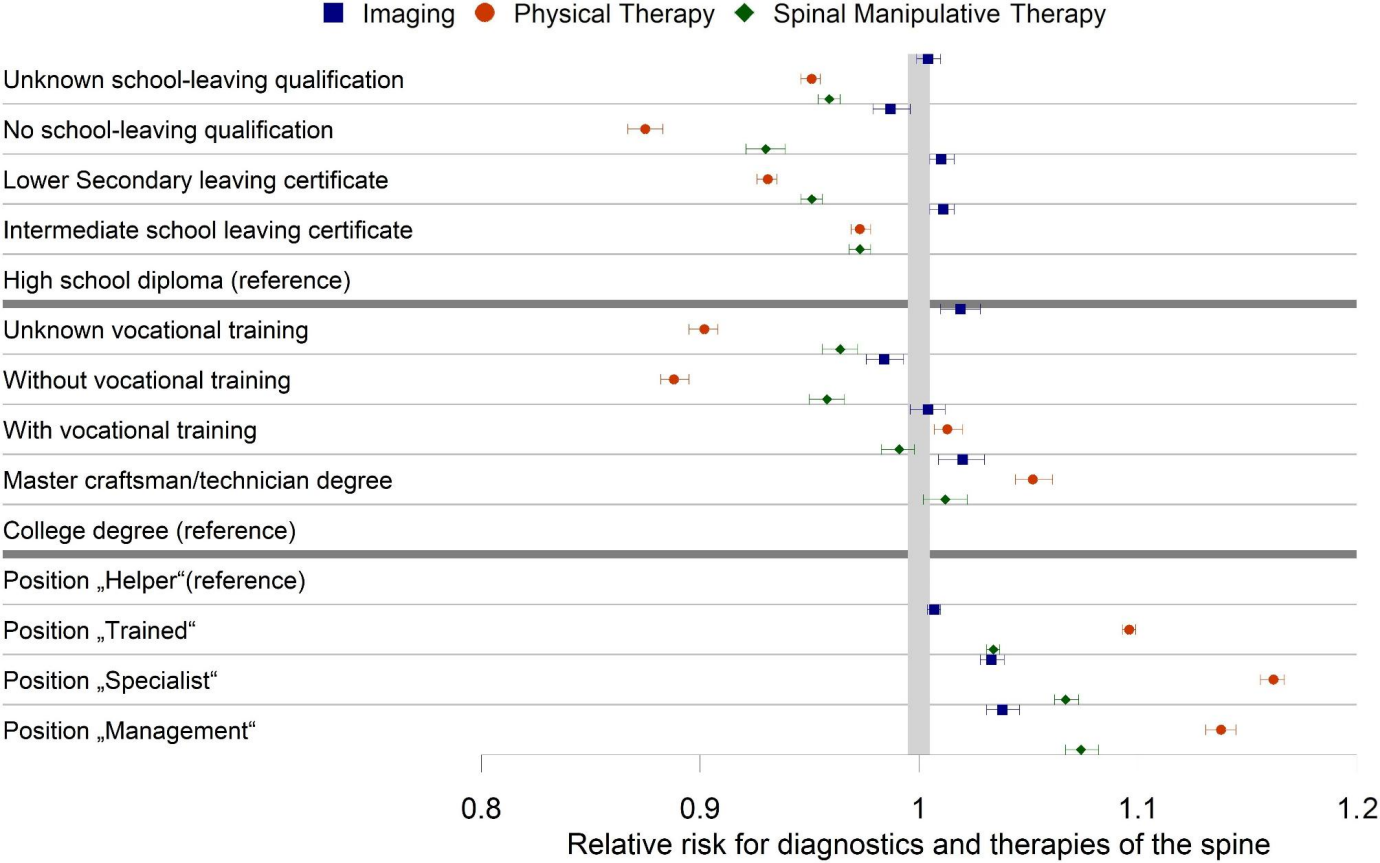
Forest plot for adjusted effect estimates with 95% confidence interval of the Poisson regression for education and occupation characteristics for diagnostic imaging and therapies for the spine based on 11.692.528 patient years of AOK members in Germany from 2012 to 2016

**Fig. 2:**
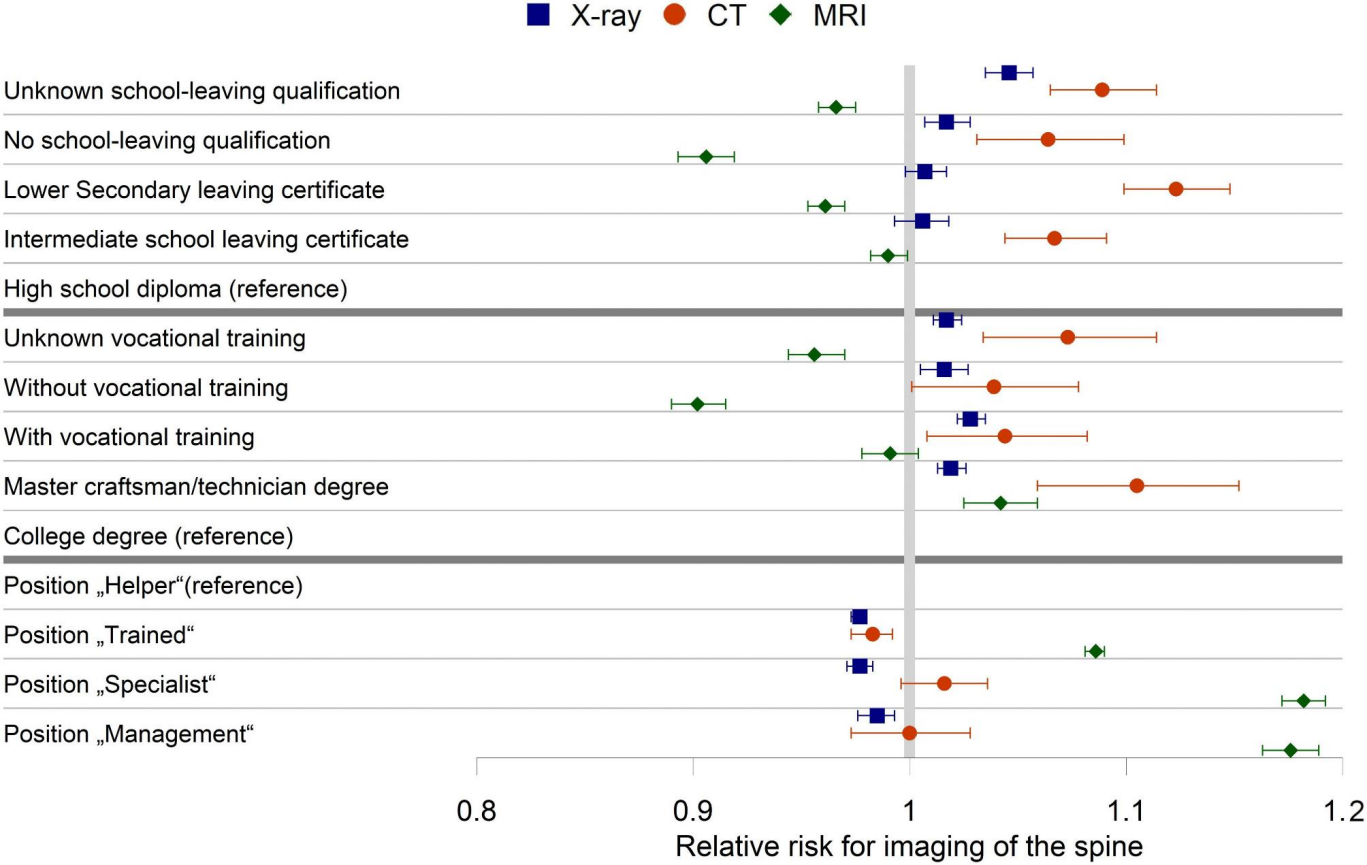
Forest plot for adjusted effect estimates with 95% confidence interval of the Poisson regression for education and occupation characteristics for diagnostic imaging of the spine based on 11.692.528 patient years of AOK members in Germany from 2012 to 2016. Abbreviations: CT- Computed tomography, MRI- magnetic resonance imaging

The differences for SES groups were small for overall diagnostic imaging and radiography. For computed tomography, there is a higher risk ratio for lower education and nearly no differences for the occupational position. The highest differences in SES variables, were found for magnetic resonance imaging. While the differences for the two highest occupational positions have been overlapping for magnetic resonance imaging, we see for magnetic resonance imaging and professional education a higher risk ratio for those with a “Master craftsman/technician” degree compared to those with a college degree (Supplementary table S5, Figs. 1 and 2).

### Regression results for non-pharmaceutical conservative therapies

In 31.7% of the patient-years physical therapy for the spine was observed. Exercise therapy was most common (18.4%), followed by manual therapy (10.7%) and massage (6.6% of the patient-years). Spinal manipulative therapy was observed in 24.5% and acupuncture in 4.0% of the patient-years (Supplementary table S4). Regression models indicated a decreased risk ratio of spinal manipulative therapy with increasing age, whereas the risk ratio of acupuncture increased with age. A consultation with an orthopedic practitioner was associated with more non-pharmaceutical conservative therapies, especially spinal manipulative therapy (RR 5.56 CI: 5.55–5.57) and acupuncture (RR 4.62 CI: 4.59–4.65) (Supplementary table S5).

**Fig. 3:**
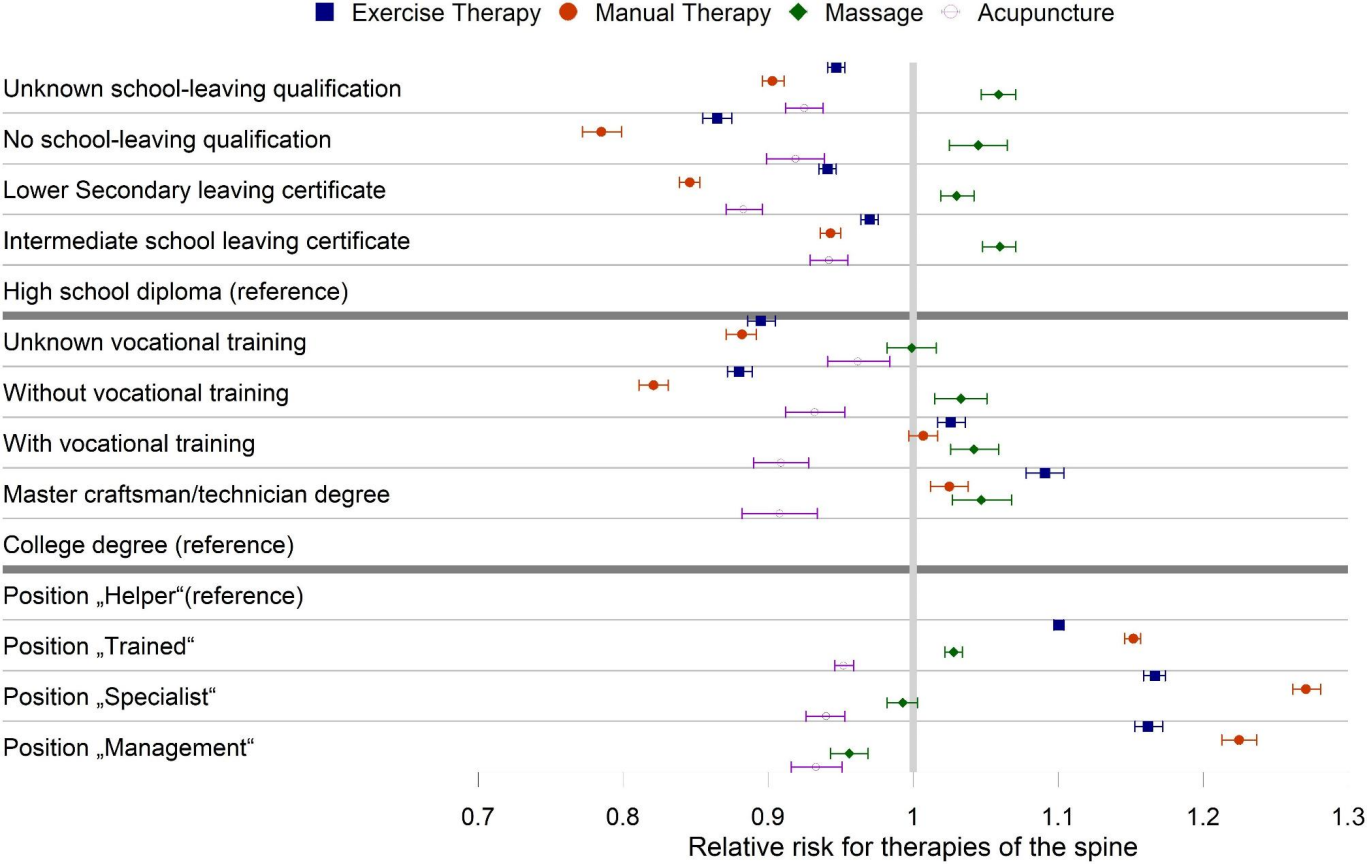
Forest plot for adjusted effect estimates with 95% confidence interval of the Poisson regression for education and occupation characteristics for spinal non-pharmaceutical conservative therapies based on 11.692.528 patient years of AOK members in Germany from 2012 to 2016

For spinal manipulative therapy, we also observed a reduced risk ratio for those with lower school education. For professional education and occupational position, we are not able to distinguish between the two highest categories. The largest effect of the SES was present for physical therapy. Here the second highest categories for professional education (master craftsman/technician degree) and occupational position (“Specialist”) had the highest risk ratio for physical therapy (Supplementary table S5, Fig. 1). High school/professional education had also a higher risk ratio for acupuncture. In contrast, a higher occupational position shows a lower likelihood for acupuncture. Within physical therapy, we see a uniformly higher risk ratio of massage among those with less education, but inconclusive results regarding occupational position. For exercise therapy and manual therapy, we see a clear trend for school education and occupational position with a higher risk ratio of utilization for a higher SES level. However, for professional education those with vocational training or master craftsman/technician degree have a higher risk ratio for those therapies compared with those without vocational training as well as those with a college degree (Supplementary table S5, Fig. 3).

## Discussion

### Main results

This study expands previous research by showing that the impact of SES on health care utilization can also be estimated in routine healthcare data. Compared to analyses using survey data, actual rather than self-reported utilization can be captured. In addition, the large number of cases in the health insurance data enables the calculation of narrower confidence intervals. However information on the size of social networks, income or assets of the patients was unavailable in this study. The SES differences here were more pronounced for more expensive diagnostics/therapies like magnetic resonance imaging or manual therapy. Patients with higher levels of education or in a higher occupational position might be more likely to articulate their wishes or convince the physician to be treated with a certain diagnostic or therapy. Also a review shows that for patients with surgery of the spine SES differences exist in outcomes of surgery in favor of higher education and income [19]. Health interventions should therefor aim at lessening the association between SES and health [20, 21].

### Comparison of the results to other studies

According to the Global Burden of Disease Study lower back pain was the leading single cause, with a share of 7.6%, of all years lived with disability (YLD) [22]. Especially people with low education and income are disproportionately affected by chronic back pain [23]. The difference for low education compared to high education for back and neck pain in Germany is reported as an age-adjusted rate ratio of 1.24 (CI 1.12–1.37) [24]. As demonstrated in this study this difference also manifests itself in the utilization of diagnostic imaging and non-pharmaceutical conservative therapies for spinal diseases.

The direction of the effect for diagnostic imaging of regional income was mixed in routine healthcare data regarding whether other comorbidities were controlled for in the study or not [7, 8]. For patients with occupational low back pain no effect of education, but an effect of household income (> 70.000 $ vs. <30.000 $ per year) for early (up to 42 days) magnetic resonance imaging was found [9]. The direction of the socioeconomic differences for non-pharmaceutical conservative therapies was similar to previous survey-based studies. Higher education and income for patients with acute or chronic low back pain resulted in a higher utilization of physical therapy and visits to a physiotherapist, a chiropractic or an acupuncturists. In contrast, no effect of education was found for pain medication [10–14].

However the analysis of therapies is limited in survey data as self-reports may suffer from recall and social-desirability bias. Nevertheless surveys are needed to complement other data. Survey data show for example that in Germany about 5.6% of men and 19% of women have unmet needs regarding healthcare access and utilization. The major problems were a waiting list or no available appointments in outpatient care, others are not been able to (co)pay for treatment, had to work or had other commitments during opening times of practices or no available service nearby [25]. Combining data from several European countries no significant effect of education on unmet needs was found, but an effect for financial strain with an odds ratio of 1.61 (CI:1.46–1.77) [26]. The financial effect was lower in a comparable study from Canada. Focusing only on the aspects of the availability of the services the effect for financial strains was 1.31 (CI: 1.17–1.47) for Europe but insignificant for Canada [26, 27].

### Special conditions of the German healthcare system

To understand the results is it important to know that paying out of pocket for the chosen diagnostic imaging and non-pharmaceutical conservative therapies is uncommon in Germany. The exception of the rule is acupuncture, which can be billed to the health insurer for spinal diseases, osteoarthritis of the knee or is paid by the patient himself for these or other indications. This might explain the unusual distribution of the SES estimates regarding utilization of acupuncture. Patients above the age of 18 years in Germany have to copay 10% of the cost of physical therapy (§ 61 German Social Code V). So financial restraints might lead to an underutilization of the prescribed therapies without the awareness of the physician. This likely results in an underestimation of the physician prescriptions in routine healthcare data. Also outpatient physicians have budgets for medications and prescriptions for physical therapy (together with occupational therapy, speech therapy and podiatry). No budgets exist in Germany for diagnostic imaging and health services of physicians. The allocation of this budget might cause an underutilization in patients with a lower SES, despite the fact that certain severe diagnoses are excluded from the budget restrictions.

### Strengths and limitations

The greatest strength of the analysis is the large sample size without recall bias. Also the new 9-digit German job role code with its more detailed classification of education and occupational position compared to the old 5-digit code [28] allowed a more precise SES approximation. This information for a large group of patients is rare in an international perspective.

Nevertheless for education, in line with another German study, about one third had unknown school education and about one fifth unknown professional education [29]. This is a limitation at the employer level, where the education might not be adequately documented, whereas the occupation is. Also, this study could not use information on patients’ income, assets, or social network size. In addition, the analysis plan prior to the remote data processing did not include testing for interactions among SES categories or models of subgroups of the population.

Patients in unemployment, retirement, maternity leave and also those 10% with a private insurance because of their work as civil servants (e.g. police), self-employment or due to high income are not covered by the study. This selection should lead to an underestimation of the size of the socioeconomic disparities. Ultimately, the results cannot clarify whether there is an oversupply with diagnostic imaging or non-pharmaceutical conservative therapies in high SES patients or an undersupply in low SES patients.

## Conclusions

Based on routine health care data, we were able to estimate the socioeconomic status in the context of health care utilization among patients with spinal diseases. We found that the observed differences in health care utilization depend on the socioeconomic status and are more pronounced for more expensive diagnostic imaging and non-pharmaceutical conservative therapies. Research identifying barriers to equitable access to health services and their relationship to SES status is needed. As a result, health care providers must initiate and monitor appropriate interventions to counteract the impact of socioeconomic status on the utilization of health care services.

### Electronic supplementary material

Below is the link to the electronic supplementary material.

Supplementary Material 1

## Data Availability

The authors confirm that the data utilized in this study cannot be made available in the manuscript, the supplemental files, or in a public repository due to German data protection laws (‘Bundesdatenschutzgesetz’, BDSG). Therefore, they are stored on a secure drive in the WIdO, to facilitate replication of the results. Generally, access to data of statutory health insurance funds for research purposes is possible only under the conditions defined in German Social Law (SGB V § 287). Requests for data access can be sent as a formal proposal specifying the recipient and purpose of the data transfer to the appropriate data protection agency. Access to the data used in this study can only be provided to external parties under the conditions of the cooperation contract of this research project and after written approval by the sickness fund. For assistance in obtaining access to the data, please contact wido@wido.bv.aok.de.

## Abbreviations

AOK: “Allgemeine Ortskrankenkassen” a statutory health insurance in Germany
CI: Confidence interval
ICD-10: International Statistical Classification of Diseases and Related Health Problems, 10th revision
NSAID: Nonsteroidal anti-inflammatory drugs
RR: Relative Risk as effect estimates in a Poisson regression model
