# Supplementary material for: Socioeconomic differences in the utilization of diagnostic imaging and non-pharmaceutical conservative therapies for spinal diseases
Source: BMC Musculoskelet Disord. 2023 Oct 2;24:774. doi: 10.1186/s12891-023-06909-6 (PMC10544477; doi:10.1186/s12891-023-06909-6)
Supplement: Supplementary file 1 — Supplementary Material 1 [file 12891_2023_6909_MOESM1_ESM.docx]

Table S1: Treatment definitions

| **Treatment** | **German Billing Code** |
| --- | --- |
|  | **Remedy position** |
| Massage therapy | X0101 – X0108 with Indication WS (Spine) |
| Exercise therapy (1 to 1) | X0301 – X0308, X0501 – X0506 with Indication WS (Spine) |
| Manual therapy | X1201 – X1204 with Indication WS (Spine) |
|  | **Outpatient code (EBM)** |
| Acupuncture | 30791 |
| Spinal manipulative therapy | 30201 |
| Outpatient Injection therapy | 30722, 30723, 30724, 30731, 02360, 34503 |
| Magnetic resonance imaging | 34411 |
| Computed tomography | 34311 |
| Radiography | 34221, 34222 |
|  | **Operation and procedure code (OPS)** |
| Inpatient Injection therapy | 8-910,8-911,8-913,8-914,8-915,8-916,8-917, ,8-919 |
| Magnetic resonance imaging | 3-802, 3-823 |
| Computer tomography | 3-203, 3-223 |

Table S2: Definition of Comorbidities

| **Diseases** | **ICD-10-GM Codes** | **Definition** |
| --- | --- | --- |
| Fractures of the spine | S12/S22/S32 | Once coded in outpatient or inpatient sector |
| Osteoarthritis (knee) | M16 | M2Q* |
| Osteoarthritis (hip) | M17 | M2Q |
| Osteoporosis | M80-82 | M2Q |
| Chronic polyarthritis | M05, M06, M08, M09 | M2Q |
| Rheumatic diseases (With typical spine involvement) | M7, M45, M46.1, M46.8, M46.9 | M2Q |
| Rheumatic diseases (Without typical spine involvement) | L94.0, L94.1, L94.3, M12.0, M12.3,M30, M31, M32, M33, M34, M35.0-6, M36.0, , | M2Q |
| Depression | F32,F33,F34.1 | M2Q |
| Anxiety disorder | F40,F41 | M2Q |
| Psychosomatic disorders | F45 | M2Q |
| Dementia | G30, G31.0, G31.82, G23.1, F00, F01, F02, F03,F05.1 | M2Q |
| Sleep disorders | G47,G25.8,F51 | M2Q |

*M2Q (At least coded in two quarters within 4 quarters with secured diagnostic certainty)

Table S3: Pain medication

| **Group** | **ATC-Code** | **Active ingredient** |
| --- | --- | --- |
| NSAID | M01AA | Butylpyrazolidines |
|  | M01AB | Acetic acid derivatives and related substances |
|  | M01AC | Oxicams |
|  | M01AE | Propionic acid derivatives |
|  | N02BA | Salicylic acid and derivatives |
| Cox-2 inhibitors | M01AH | Coxibe |
| Non-opioid analgesics | N02BB | Pyrazolones |
|  | N02BE | Anilides |
|  | N02BG | Other analgesics and antipyretics |
| weak-acting opioids | N02AA08 | Dihydrocodeine |
|  | N02AA57 | Ethyl morphine, combinations |
|  | N02AA58 | Dihydrocodeine, combinations |
|  | N02AA59 | Codeine, comb. excl. psycholeptics |
|  | N02AA65 | Codeine and diclofenac |
|  | N02AA66 | Codeine and acetylsalicylic acid |
|  | N02AA69 | Codeine and paracetamol |
|  | N02AA79 | Codeine, combinations with psycholeptics |
|  | N02AX01 | Tilidine |
|  | N02AX02 | Tramadol |
|  | N02AX05 | Meptazinol |
|  | N02AX51 | Tilidine and naloxone |
|  | N02AX53 | Tramadol and dexketoprofen |
|  | N02AX62 | Tramadol and paracetamol |
| strong-acting opioids | N01AH03 | Sufentanil |
|  | N02AA01 | Morphine |
|  | N02AA02 | Opium |
|  | N02AA03 | Hydromorphone |
|  | N02AA04 | Nicomorphine |
|  | N02AA05 | Oxycodone |
|  | N02AA51 | Morphine, combinations |
|  | N02AA55 | Oxycodone combinations |
|  | N02AB02 | Pethidine |
|  | N02AB03 | Fentanyl |
|  | N02AB52 | Pethidine, comb. excl. psycholeptics |
|  | N02AB72 | Pethidine, combinations with psycholeptics |
|  | N02AC03 | Piritramide |
|  | N02AC06 | Levomethadone |
|  | N02AC52 | Methadone, comb. excl. psycholeptics |
|  | N02AD01 | Pentazocine |
|  | N02AD02 | Phenazocine |
|  | N02AE01 | Buprenorphine |
|  | N02AF02 | Nalbufin |
|  | N02AG01 | Morphine with spasmolytics |
|  | N02AG02 | Ketobemidone with spasmolytics |
|  | N02AG03 | Pethidine with spasmolytics |
|  | N02AG04 | Hydromorphone with spasmolytics |
|  | N02AX03 | Dezocin |
|  | N02AX06 | Tapentadol |

Table S4 Description of patients with spinal diseases, spinal imaging or spinal therapies from 2012 to 2016. Patients with fractures of the spine were exclude

|  | Spinal diseases | | Diagnostic Imaging | | X-ray | | MRI | | CT | | SMT (Physician) | | PT for the spine | | ET for the spine | | MT for the spine | | MA of the spine | | ACU | |
| --- | --- | --- | --- | --- | --- | --- | --- | --- | --- | --- | --- | --- | --- | --- | --- | --- | --- | --- | --- | --- | --- | --- |
|  | Patient years | % | Patient years | % | Patient years | % | Patient years | % | Patient years | % | Patient years | % | Patient years | % | Patient years | % | Patient years | % | Patient years | % | Patient years | % |
| **Total** | 11,692,528 | 100 | 3,073,903 | 100 | 2,173,170 | 100 | 1,240,288 | 100 | 255,872 | 100 | 2,863,906 | 100 | 3,702,735 | 100 | 2,147,540 | 100 | 1,246,997 | 100 | 769,755 | 100 | 471,428 | 100 |
| ***Sociodemographic*** |  |  |  |  |  |  |  |  |  |  |  |  |  |  |  |  |  |  |  |  |  |  |
| Male | 5830821 | 49,9 | 1516348 | 49,3 | 1063078 | 48,9 | 614633 | 49,6 | 144100 | 56,3 | 1326367 | 46,3 | 1494136 | 40,4 | 913112 | 42,5 | 441659 | 35,4 | 281212 | 36,5 | 191530 | 40,6 |
| Female | 5861707 | 50,1 | 1557555 | 50,7 | 1110092 | 51,1 | 625655 | 50,4 | 111772 | 43,7 | 1537539 | 53,7 | 2208599 | 59,6 | 1234428 | 57,5 | 805338 | 64,6 | 488543 | 63,5 | 279898 | 59,4 |
| Age group 20-24 | 607412 | 5,2 | 140992 | 4,6 | 115566 | 5,3 | 39158 | 3,2 | 4619 | 1,8 | 167256 | 5,8 | 137234 | 3,7 | 85033 | 4,0 | 40241 | 3,2 | 24543 | 3,2 | 10185 | 2,2 |
| Age group 25-29 | 857852 | 7,3 | 210765 | 6,9 | 162183 | 7,5 | 72799 | 5,9 | 8679 | 3,4 | 241827 | 8,4 | 233752 | 6,3 | 135607 | 6,3 | 81774 | 6,6 | 43461 | 5,6 | 19686 | 4,2 |
| Age group 30-34 | 932688 | 8,0 | 236716 | 7,7 | 174040 | 8,0 | 90609 | 7,3 | 12709 | 5,0 | 257584 | 9,0 | 274595 | 7,4 | 157277 | 7,3 | 97440 | 7,8 | 52539 | 6,8 | 25169 | 5,3 |
| Age group 35-39 | 1009670 | 8,6 | 265142 | 8,6 | 188456 | 8,7 | 107897 | 8,7 | 17926 | 7,0 | 265764 | 9,3 | 310510 | 8,4 | 178048 | 8,3 | 108465 | 8,7 | 61874 | 8,0 | 32231 | 6,8 |
| Age group 40-44 | 1314245 | 11,2 | 356991 | 11,6 | 248726 | 11,4 | 150060 | 12,1 | 28707 | 11,2 | 338245 | 11,8 | 426716 | 11,5 | 246814 | 11,5 | 145433 | 11,7 | 89852 | 11,7 | 48943 | 10,4 |
| Age group 45-49 | 1818218 | 15,6 | 498534 | 16,2 | 342001 | 15,7 | 214436 | 17,3 | 44698 | 17,5 | 452550 | 15,8 | 613521 | 16,6 | 356250 | 16,6 | 209860 | 16,8 | 129853 | 16,9 | 76327 | 16,2 |
| Age group 50-54 | 2066724 | 17,7 | 565287 | 18,4 | 387021 | 17,8 | 241502 | 19,5 | 54722 | 21,4 | 489940 | 17,1 | 703109 | 19,0 | 408758 | 19,0 | 238409 | 19,1 | 149548 | 19,4 | 96379 | 20,4 |
| Age group 55-59 | 1874567 | 16,0 | 500016 | 16,3 | 344799 | 15,9 | 206929 | 16,7 | 51815 | 20,3 | 411398 | 14,4 | 623590 | 16,8 | 361353 | 16,8 | 204957 | 16,4 | 134293 | 17,4 | 97342 | 20,6 |
| Age group 60-64 | 1211152 | 10,4 | 299460 | 9,7 | 210378 | 9,7 | 116898 | 9,4 | 31997 | 12,5 | 239342 | 8,4 | 379708 | 10,3 | 218400 | 10,2 | 120418 | 9,7 | 83792 | 10,9 | 65166 | 13,8 |
| ***Comorbidities*** |  |  |  |  |  |  |  |  |  |  |  |  |  |  |  |  |  |  |  |  |  |  |
| Osteoarthritis (knee) | 790966 | 6,8 | 210877 | 6,9 | 142766 | 6,6 | 91458 | 7,4 | 21124 | 8,3 | 192469 | 6,7 | 269036 | 7,3 | 160146 | 7,5 | 84140 | 6,7 | 58325 | 7,6 | 88875 | 18,9 |
| Osteoarthritis (hip) | 403515 | 3,5 | 132667 | 4,3 | 92400 | 4,3 | 58633 | 4,7 | 13668 | 5,3 | 112151 | 3,9 | 151965 | 4,1 | 94112 | 4,4 | 48551 | 3,9 | 28537 | 3,7 | 29559 | 6,3 |
| Osteoporosis | 181882 | 1,6 | 57812 | 1,9 | 41748 | 1,9 | 23608 | 1,9 | 5351 | 2,1 | 48872 | 1,7 | 73052 | 2,0 | 42820 | 2,0 | 24934 | 2,0 | 15650 | 2,0 | 13519 | 2,9 |
| Chronic polyarthritis | 137829 | 1,2 | 37673 | 1,2 | 23782 | 1,1 | 20035 | 1,6 | 2836 | 1,1 | 27413 | 1,0 | 50285 | 1,4 | 31842 | 1,5 | 16901 | 1,4 | 8069 | 1,0 | 5952 | 1,3 |
| Rheumatic diseases (With typical spine involvement) | 185862 | 1,6 | 50089 | 1,6 | 32439 | 1,5 | 23657 | 1,9 | 4500 | 1,8 | 40073 | 1,4 | 66530 | 1,8 | 37534 | 1,7 | 23804 | 1,9 | 14256 | 1,9 | 9643 | 2,0 |
| Rheumatic diseases (Without typical spine involvement) | 83690 | 0,7 | 23617 | 0,8 | 15526 | 0,7 | 10727 | 0,9 | 2108 | 0,8 | 20014 | 0,7 | 32026 | 0,9 | 18726 | 0,9 | 11018 | 0,9 | 6629 | 0,9 | 4897 | 1,0 |
| Depression | 1696999 | 14,5 | 458047 | 14,9 | 304702 | 14,0 | 206607 | 16,7 | 42466 | 16,6 | 407704 | 14,2 | 589340 | 15,9 | 347466 | 16,2 | 193368 | 15,5 | 128340 | 16,7 | 95168 | 20,2 |
| Anxiety disorder | 580462 | 5,0 | 153798 | 5,0 | 105287 | 4,8 | 66226 | 5,3 | 13429 | 5,2 | 148437 | 5,2 | 210076 | 5,7 | 119579 | 5,6 | 74795 | 6,0 | 44913 | 5,8 | 29653 | 6,3 |
| Psychosomatic disorders | 1157579 | 9,9 | 331657 | 10,8 | 218482 | 10,1 | 156678 | 12,6 | 30133 | 11,8 | 319615 | 11,2 | 445016 | 12,0 | 253737 | 11,8 | 164881 | 13,2 | 93840 | 12,2 | 76991 | 16,3 |
| Sleep disorders | 649749 | 5,6 | 175618 | 5,7 | 114690 | 5,3 | 80319 | 6,5 | 18082 | 7,1 | 147102 | 5,1 | 212896 | 5,7 | 126833 | 5,9 | 68151 | 5,5 | 44599 | 5,8 | 36304 | 7,7 |
| **Diagnoses M40-54** |  |  |  |  |  |  |  |  |  |  |  |  |  |  |  |  |  |  |  |  |  |  |
| 1 Quarter | 4204412 | 36,0 | 858666 | 27,9 | 676218 | 31,1 | 224749 | 18,1 | 52844 | 20,7 | 912371 | 31,9 | 961614 | 26,0 | 507883 | 23,6 | 325788 | 26,1 | 210140 | 27,3 | 69323 | 14,7 |
| 2 Quarters | 2534749 | 21,7 | 756354 | 24,6 | 547647 | 25,2 | 296517 | 23,9 | 57675 | 22,5 | 686125 | 24,0 | 849758 | 22,9 | 489880 | 22,8 | 281357 | 22,6 | 169375 | 22,0 | 101945 | 21,6 |
| 3 Quarters | 1941722 | 16,6 | 585360 | 19,0 | 397314 | 18,3 | 274301 | 22,1 | 52826 | 20,6 | 514688 | 18,0 | 712090 | 19,2 | 426651 | 19,9 | 239568 | 19,2 | 143937 | 18,7 | 104776 | 22,2 |
| 4 Quarters | 3007494 | 25,7 | 872310 | 28,4 | 551838 | 25,4 | 443780 | 35,8 | 92331 | 36,1 | 750475 | 26,2 | 1178749 | 31,8 | 722851 | 33,7 | 400086 | 32,1 | 246220 | 32,0 | 195378 | 41,4 |
| **Physician consultations** |  |  |  |  |  |  |  |  |  |  |  |  |  |  |  |  |  |  |  |  |  |  |
| General practitioner | 8769549 | 75,0 | 2162823 | 70,4 | 1489168 | 68,5 | 914196 | 73,7 | 201040 | 78,6 | 2026477 | 70,8 | 2792756 | 75,4 | 1604609 | 74,7 | 942155 | 75,6 | 607522 | 78,9 | 354058 | 75,1 |
| Orthopedist practitioner | 3683485 | 31,5 | 2030181 | 66,0 | 1594068 | 73,4 | 761129 | 61,4 | 136063 | 53,2 | 1961602 | 68,5 | 1720927 | 46,5 | 1106319 | 51,5 | 562917 | 45,1 | 302535 | 39,3 | 336503 | 71,4 |
| Surgeon | 622372 | 5,3 | 339325 | 11,0 | 256697 | 11,8 | 151357 | 12,2 | 24538 | 9,6 | 187170 | 6,5 | 255201 | 6,9 | 168460 | 7,8 | 80446 | 6,5 | 42517 | 5,5 | 35464 | 7,5 |
| Preventive and rehabilitation physician | 264136 | 2,3 | 123096 | 4,0 | 82454 | 3,8 | 59861 | 4,8 | 11000 | 4,3 | 138166 | 4,8 | 153574 | 4,1 | 93412 | 4,3 | 62047 | 5,0 | 22559 | 2,9 | 29622 | 6,3 |
| **Pain medication** |  |  |  |  |  |  |  |  |  |  |  |  |  |  |  |  |  |  |  |  |  |  |
| NSAIDs 0.1 to 30 DDD | 3191724 | 27,3 | 897224 | 29,2 | 667802 | 30,7 | 325658 | 26,3 | 67653 | 26,4 | 838943 | 29,3 | 975742 | 26,4 | 571333 | 26,6 | 318176 | 25,5 | 192833 | 25,1 | 120620 | 25,6 |
| NSAIDs 30 to 90 DDD | 1986221 | 17,0 | 684067 | 22,3 | 462717 | 21,3 | 317158 | 25,6 | 68847 | 26,9 | 553931 | 19,3 | 720322 | 19,5 | 441848 | 20,6 | 230793 | 18,5 | 139523 | 18,1 | 102894 | 21,8 |
| NSAIDs 90 to 180 DDD | 506001 | 4,3 | 182737 | 5,9 | 114151 | 5,3 | 98146 | 7,9 | 22138 | 8,7 | 135430 | 4,7 | 194799 | 5,3 | 124304 | 5,8 | 59730 | 4,8 | 39163 | 5,1 | 33018 | 7,0 |
| NSAIDs 180+ DDD | 251021 | 2,1 | 82246 | 2,7 | 49489 | 2,3 | 44303 | 3,6 | 11343 | 4,4 | 57405 | 2,0 | 88322 | 2,4 | 57718 | 2,7 | 25150 | 2,0 | 17983 | 2,3 | 16316 | 3,5 |
| Cox-2 inhibitors 0.1 to 30 DDD | 232309 | 2,0 | 86079 | 2,8 | 56010 | 2,6 | 1154554 | 93,1 | 9054 | 3,5 | 67376 | 2,4 | 95955 | 2,6 | 54786 | 2,6 | 37195 | 3,0 | 17906 | 2,3 | 12250 | 2,6 |
| Cox-2 inhibitors 30 to 90 DDD | 102231 | 0,9 | 41307 | 1,3 | 24460 | 1,1 | 43260 | 3,5 | 4827 | 1,9 | 29326 | 1,0 | 47125 | 1,3 | 27057 | 1,3 | 19099 | 1,5 | 8693 | 1,1 | 6357 | 1,3 |
| Cox-2 inhibitors 90 to 180 DDD | 45261 | 0,4 | 18010 | 0,6 | 10178 | 0,5 | 24020 | 1,9 | 2078 | 0,8 | 12329 | 0,4 | 21367 | 0,6 | 12542 | 0,6 | 8751 | 0,7 | 3942 | 0,5 | 3010 | 0,6 |
| Cox-2 inhibitors 180+ DDD | 35259 | 0,3 | 12251 | 0,4 | 6693 | 0,3 | 11084 | 0,9 | 1433 | 0,6 | 8158 | 0,3 | 15692 | 0,4 | 9263 | 0,4 | 6197 | 0,5 | 2880 | 0,4 | 2134 | 0,5 |
| Non-opioid analgesics 0.1 to 30 DDD | 2056106 | 17,6 | 712896 | 23,2 | 476257 | 21,9 | 340528 | 27,5 | 72518 | 28,3 | 561678 | 19,6 | 748020 | 20,2 | 455047 | 21,2 | 248714 | 19,9 | 142112 | 18,5 | 105765 | 22,4 |
| Non-opioid analgesics 30 to 90 DDD | 131710 | 1,1 | 59125 | 1,9 | 33599 | 1,5 | 36343 | 2,9 | 8623 | 3,4 | 38432 | 1,3 | 62415 | 1,7 | 40693 | 1,9 | 21735 | 1,7 | 11496 | 1,5 | 10638 | 2,3 |
| Non-opioid analgesics 90 to 180 DDD | 19540 | 0,2 | 8183 | 0,3 | 4396 | 0,2 | 5128 | 0,4 | 1289 | 0,5 | 5122 | 0,2 | 9299 | 0,3 | 6262 | 0,3 | 3236 | 0,3 | 1603 | 0,2 | 1708 | 0,4 |
| Non-opioid analgesics 180+ DDD | 7139 | 0,1 | 2710 | 0,1 | 1413 | 0,1 | 1624 | 0,1 | 421 | 0,2 | 1858 | 0,1 | 3329 | 0,1 | 2118 | 0,1 | 1222 | 0,1 | 647 | 0,1 | 524 | 0,1 |
| Weak opioids 0.1 to 30 DDD | 428176 | 3,7 | 213725 | 7,0 | 128537 | 5,9 | 126166 | 10,2 | 29702 | 11,6 | 141075 | 4,9 | 189085 | 5,1 | 128628 | 6,0 | 58756 | 4,7 | 29285 | 3,8 | 30783 | 6,5 |
| Weak opioids 30 to 90 DDD | 91223 | 0,8 | 46303 | 1,5 | 24515 | 1,1 | 31183 | 2,5 | 7730 | 3,0 | 26668 | 0,9 | 42530 | 1,1 | 29006 | 1,4 | 13733 | 1,1 | 6843 | 0,9 | 7508 | 1,6 |
| Weak opioids 90 to 180 DDD | 33562 | 0,3 | 13725 | 0,4 | 7164 | 0,3 | 8774 | 0,7 | 2252 | 0,9 | 8379 | 0,3 | 13827 | 0,4 | 9203 | 0,4 | 4497 | 0,4 | 2359 | 0,3 | 2580 | 0,5 |
| Weak opioids 180+ DDD | 47691 | 0,4 | 14801 | 0,5 | 7712 | 0,4 | 8896 | 0,7 | 2226 | 0,9 | 9732 | 0,3 | 15210 | 0,4 | 9565 | 0,4 | 5041 | 0,4 | 2662 | 0,3 | 2850 | 0,6 |
| Strong opioids 0.1 to 30 DDD | 43501 | 0,4 | 24675 | 0,8 | 11622 | 0,5 | 17935 | 1,4 | 4501 | 1,8 | 12943 | 0,5 | 21705 | 0,6 | 15219 | 0,7 | 6989 | 0,6 | 3295 | 0,4 | 3829 | 0,8 |
| Strong opioids 30 to 90 DDD | 13418 | 0,1 | 6511 | 0,2 | 3003 | 0,1 | 4541 | 0,4 | 1276 | 0,5 | 3459 | 0,1 | 6339 | 0,2 | 4229 | 0,2 | 2275 | 0,2 | 1031 | 0,1 | 1185 | 0,3 |
| Strong opioids 90 to 180 DDD | 8952 | 0,1 | 3590 | 0,1 | 1700 | 0,1 | 2325 | 0,2 | 686 | 0,3 | 2086 | 0,1 | 3694 | 0,1 | 2299 | 0,1 | 1395 | 0,1 | 555 | 0,1 | 649 | 0,1 |
| Strong opioids 180+ DDD | 13993 | 0,1 | 4755 | 0,2 | 2187 | 0,1 | 2989 | 0,2 | 913 | 0,4 | 2667 | 0,1 | 4783 | 0,1 | 2945 | 0,1 | 1684 | 0,1 | 801 | 0,1 | 827 | 0,2 |
| **Sick days leave** |  |  |  |  |  |  |  |  |  |  |  |  |  |  |  |  |  |  |  |  |  |  |
| No | 7322939 | 62,6 | 1539619 | 50,1 | 1141575 | 52,5 | 530306 | 42,8 | 94367 | 36,9 | 1636135 | 57,1 | 2161574 | 58,4 | 1201714 | 56,0 | 754531 | 60,5 | 478866 | 62,2 | 251013 | 53,2 |
| 1 to 7 days | 1743786 | 14,9 | 400823 | 13,0 | 302414 | 13,9 | 132810 | 10,7 | 25749 | 10,1 | 436545 | 15,2 | 445479 | 12,0 | 257341 | 12,0 | 144356 | 11,6 | 88494 | 11,5 | 49023 | 10,4 |
| 8 to 21 days | 1473179 | 12,6 | 497134 | 16,2 | 353648 | 16,3 | 195365 | 15,8 | 43940 | 17,2 | 401688 | 14,0 | 511579 | 13,8 | 294737 | 13,7 | 168817 | 13,5 | 103477 | 13,4 | 68536 | 14,5 |
| 22 to 42 days | 603413 | 5,2 | 297918 | 9,7 | 187722 | 8,6 | 154483 | 12,5 | 37257 | 14,6 | 194086 | 6,8 | 277161 | 7,5 | 173270 | 8,1 | 87968 | 7,1 | 50964 | 6,6 | 44983 | 9,5 |
| 42+ days | 549211 | 4,7 | 338409 | 11,0 | 187811 | 8,6 | 227324 | 18,3 | 54559 | 21,3 | 1636135 | 57,1 | 306942 | 8,3 | 220478 | 10,3 | 91325 | 7,3 | 47954 | 6,2 | 57873 | 12,3 |
| **Education** |  |  |  |  |  |  |  |  |  |  |  |  |  |  |  |  |  |  |  |  |  |  |
| Unknown school-leaving qualification | 3987669 | 34,1 | 1069787 | 34,8 | 758771 | 34,9 | 426248 | 34,4 | 92557 | 36,2 | 958860 | 33,5 | 1180698 | 31,9 | 702850 | 32,7 | 358311 | 28,7 | 255783 | 33,2 | 183790 | 39,0 |
| No school-leaving qualification | 307915 | 2,6 | 76179 | 2,5 | 54228 | 2,5 | 29332 | 2,4 | 7257 | 2,8 | 63553 | 2,2 | 69836 | 1,9 | 43188 | 2,0 | 16878 | 1,4 | 16410 | 2,1 | 13659 | 2,9 |
| Lower Secondary leaving certificate | 3477109 | 29,7 | 939712 | 30,6 | 662348 | 30,5 | 379591 | 30,6 | 90222 | 35,3 | 824836 | 28,8 | 1050894 | 28,4 | 673002 | 31,3 | 284622 | 22,8 | 213476 | 27,7 | 137329 | 29,1 |
| Intermediate school leaving certificate | 2894126 | 24,8 | 754657 | 24,6 | 531469 | 24,5 | 312435 | 25,2 | 53262 | 20,8 | 751871 | 26,3 | 1067715 | 28,8 | 546917 | 25,5 | 454126 | 36,4 | 227361 | 29,5 | 100601 | 21,3 |
| High school diploma | 1025708 | 8,8 | 233568 | 7,6 | 166354 | 7,7 | 92682 | 7,5 | 12574 | 4,9 | 264786 | 9,2 | 333592 | 9,0 | 181583 | 8,5 | 133060 | 10,7 | 56725 | 7,4 | 36049 | 7,6 |
| Unknown vocational training | 2453842 | 21,0 | 661808 | 21,5 |  |  | 259077 | 20,9 | 56676 | 22,2 | 584665 | 20,4 | 676364 | 18,3 | 402065 | 18,7 | 198502 | 15,9 | 150101 | 19,5 | 114091 | 24,2 |
| Without vocational training | 2021184 | 17,3 | 515127 | 16,8 | 371619 | 17,1 | 194455 | 15,7 | 46557 | 18,2 | 449010 | 15,7 | 508325 | 13,7 | 322282 | 15,0 | 122725 | 9,8 | 114247 | 14,8 | 92236 | 19,6 |
| With vocational training | 6464084 | 55,3 | 1729717 | 56,3 | 1211476 | 55,7 | 717862 | 57,9 | 142231 | 55,6 | 1645247 | 57,4 | 2257624 | 61,0 | 1293620 | 60,2 | 810608 | 65,0 | 459443 | 59,7 | 242362 | 51,4 |
| Master craftsman/technician degree | 353674 | 3,0 | 77880 | 2,5 | 53668 | 2,5 | 32529 | 2,6 | 5956 | 2,3 | 80434 | 2,8 | 118612 | 3,2 | 60477 | 2,8 | 49445 | 4,0 | 21815 | 2,8 | 9011 | 1,9 |
| College degree | 399743 | 3,4 | 89371 | 2,9 | 63006 | 2,9 | 36365 | 2,9 | 4452 | 1,7 | 104550 | 3,7 | 141810 | 3,8 | 69096 | 3,2 | 65717 | 5,3 | 24149 | 3,1 | 13728 | 2,9 |
| **Occupation** |  |  |  |  |  |  |  |  |  |  |  |  |  |  |  |  |  |  |  |  |  |  |
| Agriculture | 256368 | 2,2 | 56392 | 1,8 | 40128 | 1,8 | 21661 | 1,7 | 5046 | 2,0 | 51950 | 1,8 | 69129 | 1,9 | 35693 | 1,7 | 25235 | 2,0 | 14384 | 1,9 | 6993 | 1,5 |
| Raw material extraction, production | 3107581 | 26,6 | 849759 | 27,6 | 605004 | 27,8 | 336688 | 27,1 | 78859 | 30,8 | 753107 | 26,3 | 920959 | 24,9 | 554055 | 25,8 | 279824 | 22,4 | 193759 | 25,2 | 125109 | 26,5 |
| Construction | 864378 | 7,4 | 224463 | 7,3 | 156043 | 7,2 | 92760 | 7,5 | 21978 | 8,6 | 195005 | 6,8 | 228851 | 6,2 | 139306 | 6,5 | 68176 | 5,5 | 43377 | 5,6 | 26748 | 5,7 |
| Natural science | 214548 | 1,8 | 47564 | 1,5 | 33348 | 1,5 | 19097 | 1,5 | 3810 | 1,5 | 47397 | 1,7 | 57967 | 1,6 | 33384 | 1,6 | 19606 | 1,6 | 10294 | 1,3 | 6469 | 1,4 |
| Transport, logistics and security | 2621758 | 22,4 | 708729 | 23,1 | 502925 | 23,1 | 280330 | 22,6 | 66870 | 26,1 | 597840 | 20,9 | 727760 | 19,7 | 433796 | 20,2 | 208596 | 16,7 | 164610 | 21,4 | 120217 | 25,5 |
| Commercial services, distribution, tourism | 1332634 | 11,4 | 352416 | 11,5 | 254121 | 11,7 | 139424 | 11,2 | 24386 | 9,5 | 351352 | 12,3 | 445396 | 12,0 | 254193 | 11,8 | 155411 | 12,5 | 94930 | 12,3 | 53395 | 11,3 |
| Business and law | 1335204 | 11,4 | 332767 | 10,8 | 234682 | 10,8 | 137544 | 11,1 | 21139 | 8,3 | 359023 | 12,5 | 519230 | 14,0 | 286459 | 13,3 | 210819 | 16,9 | 100756 | 13,1 | 50003 | 10,6 |
| Health, social services, teaching and education | 1797835 | 15,4 | 466949 | 15,2 | 322179 | 14,8 | 199066 | 16,0 | 31713 | 12,4 | 468277 | 16,4 | 684271 | 18,5 | 385756 | 18,0 | 259092 | 20,8 | 138650 | 18,0 | 77641 | 16,5 |
| Language, media, art, culture and design | 161278 | 1,4 | 34626 | 1,1 | 24575 | 1,1 | 13641 | 1,1 | 2043 | 0,8 | 39751 | 1,4 | 48924 | 1,3 | 24774 | 1,2 | 20147 | 1,6 | 8941 | 1,2 | 4812 | 1,0 |
| Military | 944 | 0,0 | 238 | 0,0 | 165 | 0,0 | 77 | 0,0 | 28 | 0,0 | 204 | 0,0 | 248 | 0,0 | 124 | 0,0 | 91 | 0,0 | 54 | 0,0 | 41 | 0,0 |
| Position „Helper“ | 3313943 | 28,3 | 870203 | 28,3 | 625879 | 28,8 | 332770 | 26,8 | 79242 | 31,0 | 751116 | 26,2 | 933048 | 25,2 | 556849 | 25,9 | 264590 | 21,2 | 214800 | 27,9 | 158650 | 33,7 |
| Position „Trained“ | 7042991 | 60,2 | 1903157 | 61,9 | 1339399 | 61,6 | 781977 | 63,0 | 156530 | 61,2 | 1794701 | 62,7 | 2332040 | 63,0 | 1359076 | 63,3 | 804678 | 64,5 | 478755 | 62,2 | 273303 | 58,0 |
| Position „Specialist“ | 857946 | 7,3 | 193167 | 6,3 | 133393 | 6,1 | 81021 | 6,5 | 13550 | 5,3 | 201048 | 7,0 | 277303 | 7,5 | 148549 | 6,9 | 109927 | 8,8 | 48744 | 6,3 | 24448 | 5,2 |
| Position „Management“ | 477647 | 4,1 | 107376 | 3,5 | 74499 | 3,4 | 44520 | 3,6 | 6550 | 2,6 | 117041 | 4,1 | 160344 | 4,3 | 83066 | 3,9 | 67802 | 5,4 | 27456 | 3,6 | 15027 | 3,2 |

Table S5 Poisson regression analysis(multiple adjusted): relative risks for spinal imaging and conservative non-pharmacological spinal therapies from 2012 to 2016 at the patient level. Effects of 96 spatial regions are not shown. Patients with fractures of the spine were excluded.

|  | Diagnostic Imaging | | | X-ray | | | MRI | | | CT | | | SMT (Physician) | | | PT for the spine | | | ET for the spine | | | MT for the spine | | | MA of the spine | | | ACU | | |
| --- | --- | --- | --- | --- | --- | --- | --- | --- | --- | --- | --- | --- | --- | --- | --- | --- | --- | --- | --- | --- | --- | --- | --- | --- | --- | --- | --- | --- | --- | --- |
|  | RR | 95 % CI | | RR | 95 % CI | | RR | 95 % CI | | RR | 95 % CI | | RR | 95 % CI | | RR | 95 % CI | | RR | 95 % CI | | RR | 95 % CI | | RR | 95 % CI | | RR | 95 % CI | |
| Year 2012 | 1(ref.) |  |  | 1(ref.) |  |  | 1(ref.) |  |  | 1(ref.) |  |  | 1 (ref.) |  |  | 1 (ref.) |  |  | 1 (ref.) |  |  | 1 (ref.) |  |  | 1(ref.) |  |  | 1(ref.) |  |  |
| Year 2013 | 0.976 | 0.972 | 0.979 | 0.962 | 0.958 | 0.965 | 1.030 | 1.024 | 1.036 | 0.897 | 0.886 | 0.907 | 0.978 | 0.975 | 0.981 | 0.996 | 0.993 | 0.999 | 1.003 | 0.999 | 1.007 | 1.057 | 1.051 | 1.063 | 0.889 | 0.883 | 0.895 | 0.953 | 0.944 | 0.961 |
| Year 2014 | 0.958 | 0.954 | 0.961 | 0.917 | 0.914 | 0.921 | 1.066 | 1.059 | 1.071 | 0.839 | 0.829 | 0.849 | 0.954 | 0.950 | 0.957 | 1.024 | 1.020 | 1.027 | 1.035 | 1.030 | 1.039 | 1.135 | 1.129 | 1.142 | 0.833 | 0.828 | 0.839 | 0.897 | 0.889 | 0.905 |
| Year 2015 | 0.928 | 0.925 | 0.931 | 0.861 | 0.857 | 0.864 | 1.106 | 1.010 | 1.112 | 0.759 | 0.749 | 0.768 | 0.928 | 0.924 | 0.931 | 1.007 | 1.004 | 1.010 | 1.028 | 1.024 | 1.033 | 1.158 | 1.151 | 1.164 | 0.732 | 0.727 | 0.737 | 0.876 | 0.868 | 0.884 |
| Year 2016 | 0.910 | 0.907 | 0.914 | 0.824 | 0.821 | 0.828 | 1.131 | 1.124 | 1.137 | 0.693 | 0.685 | 0.702 | 0.914 | 0.911 | 0.917 | 0.995 | 0.992 | 0.998 | 1.023 | 1.019 | 1.027 | 1.187 | 1.181 | 1.193 | 0.649 | 0.644 | 0.653 | 0.846 | 0.838 | 0.853 |
| ***Sociodemographic*** |  |  |  |  |  |  |  |  |  |  |  |  |  |  |  |  |  |  |  |  |  |  |  |  |  |  |  |  |  |  |
| Female (Ref. Male) | 0.995 | 0.992 | 0.998 | 1.002 | 0.999 | 1.005 | 0.981 | 0.977 | 0.986 | 0.861 | 0.852 | 0.869 | 1.063 | 1.060 | 1.066 | 1.396 | 1.392 | 1.399 | 1.322 | 1.317 | 1.326 | 1.474 | 1.468 | 1.481 | 1.682 | 1.672 | 1.692 | 1.343 | 1.334 | 1.353 |
| Age group 20-24 | 1(ref.) |  |  | 1(ref.) |  |  | 1(ref.) |  |  | 1(ref.) |  |  | 1 (ref.) |  |  | 1 (ref.) |  |  | 1 (ref.) |  |  | 1 (ref.) |  |  | 1(ref.) |  |  | 1(ref.) |  |  |
| Age group 25-29 | 1.008 | 1.001 | 1.014 | 0.964 | 0.957 | 0.971 | 1.179 | 1.164 | 1.193 | 1.261 | 1.216 | 1.307 | 0.992 | 0.986 | 0.998 | 1.146 | 1.139 | 1.154 | 1.089 | 1.080 | 1.099 | 1.236 | 1.221 | 1.250 | 1.244 | 1.225 | 1.263 | 1.301 | 1.270 | 1.332 |
| Age group 30-34 | 1.021 | 1.014 | 1.028 | 0.944 | 0.937 | 0.951 | 1.283 | 1.267 | 1.298 | 1.593 | 1.540 | 1.648 | 0.972 | 0.967 | 0.978 | 1.242 | 1.235 | 1.250 | 1.164 | 1.154 | 1.173 | 1.353 | 1.338 | 1.369 | 1.403 | 1.382 | 1.424 | 1.487 | 1.453 | 1.522 |
| Age group 35-39 | 1.025 | 1.018 | 1.031 | 0.927 | 0.921 | 0.934 | 1.315 | 1.299 | 1.330 | 1.905 | 1.844 | 1.968 | 0.923 | 0.918 | 0.929 | 1.278 | 1.270 | 1.286 | 1.183 | 1.173 | 1.192 | 1.397 | 1.382 | 1.413 | 1.504 | 1.482 | 1.526 | 1.639 | 1.602 | 1.676 |
| Age group 40-44 | 1.010 | 1.004 | 1.017 | 0.902 | 0.895 | 0.908 | 1.305 | 1.291 | 1.320 | 2.118 | 2.052 | 2.185 | 0.876 | 0.871 | 0.881 | 1.302 | 1.295 | 1.310 | 1.199 | 1.190 | 1.209 | 1.420 | 1.404 | 1.435 | 1.564 | 1.542 | 1.586 | 1.715 | 1.678 | 1.752 |
| Age group 45-49 | 0.988 | 0.982 | 0.994 | 0.880 | 0.874 | 0.886 | 1.257 | 1.243 | 1.271 | 2.245 | 2.178 | 2.316 | 0.827 | 0.822 | 0.831 | 1.292 | 1.284 | 1.299 | 1.191 | 1.183 | 1.200 | 1.391 | 1.376 | 1.406 | 1.555 | 1.533 | 1.576 | 1.761 | 1.724 | 1.799 |
| Age group 50-54 | 0.970 | 0.965 | 0.976 | 0.874 | 0.868 | 0.880 | 1.188 | 1.176 | 1.202 | 2.337 | 2.266 | 2.409 | 0.777 | 0.773 | 0.782 | 1.262 | 1.255 | 1.269 | 1.171 | 1.163 | 1.180 | 1.320 | 1.306 | 1.334 | 1.536 | 1.515 | 1.557 | 1.807 | 1.770 | 1.845 |
| Age group 55-59 | 0.944 | 0.938 | 0.950 | 0.870 | 0.864 | 0.876 | 1.086 | 1.074 | 1.098 | 2.367 | 2.295 | 2.441 | 0.724 | 0.720 | 0.728 | 1.215 | 1.208 | 1.222 | 1.125 | 1.116 | 1.133 | 1.231 | 1.217 | 1.244 | 1.513 | 1.492 | 1.534 | 1.851 | 1.812 | 1.890 |
| Age group 60-64 | 0.906 | 0.900 | 0.912 | 0.864 | 0.858 | 0.871 | 0.955 | 0.944 | 0.967 | 2.268 | 2.198 | 2.341 | 0.681 | 0.676 | 0.685 | 1.171 | 1.164 | 1.179 | 1.076 | 1.067 | 1.085 | 1.157 | 1.144 | 1.171 | 1.510 | 1.488 | 1.533 | 1.849 | 1.809 | 1.889 |
| ***Comorbidities*** |  |  |  |  |  |  |  |  |  |  |  |  |  |  |  |  |  |  |  |  |  |  |  |  |  |  |  |  |  |  |
| Osteoarthritis (knee) | 0.855 | 0.851 | 0.859 | 0.851 | 0.846 | 0.856 | 0.833 | 0.827 | 0.839 | 0.859 | 0.847 | 0.872 | 0.952 | 0.948 | 0.957 | 0.941 | 0.937 | 0.945 | 0.927 | 0.922 | 0.932 | 0.900 | 0.894 | 0.907 | 1.025 | 1.016 | 1.035 | 2.162 | 2.145 | 2.179 |
| Osteoarthritis (hip) | 0.983 | 0.977 | 0.988 | 1.006 | 1.000 | 1.013 | 0.961 | 0.959 | 0.976 | 0.996 | 0.978 | 1.014 | 0.992 | 0.986 | 0.998 | 0.974 | 0.969 | 0.979 | 1.002 | 0.995 | 1.008 | 0.928 | 0.919 | 0.936 | 1.025 | 1.016 | 1.035 | 1.019 | 1.007 | 1.031 |
| Osteoporosis | 1.035 | 1.027 | 1.044 | 1.081 | 1.070 | 1.092 | 0.981 | 0.968 | 0.994 | 1.053 | 1.024 | 1.082 | 1.007 | 0.998 | 1.016 | 0.997 | 0.989 | 1.004 | 1.023 | 1.013 | 1.033 | 0.966 | 0.954 | 0.978 | 1.015 | 0.999 | 1.031 | 1.023 | 1.005 | 1.041 |
| Chronic polyarthritis | 1.008 | 0.999 | 1.017 | 1.000 | 0.989 | 1.011 | 1.011 | 0.998 | 1.025 | 0.950 | 0.922 | 0.979 | 0.955 | 0.945 | 0.964 | 0.983 | 0.975 | 0.991 | 0.972 | 0.962 | 0.982 | 0.994 | 0.981 | 1.008 | 1.016 | 0.999 | 1.034 | 0.926 | 0.907 | 0.946 |
| Rheumatic diseases (With typical spine involvement) | 1.012 | 1.002 | 1.023 | 1.026 | 1.012 | 1.039 | 1.027 | 1.012 | 1.042 | 0.757 | 0.729 | 0.786 | 0.860 | 0.850 | 0.871 | 1.028 | 1.019 | 1.037 | 1.103 | 1.091 | 1.116 | 0.978 | 0.963 | 0.994 | 0.845 | 0.826 | 0.864 | 0.859 | 0.837 | 0.882 |
| Rheumatic diseases (Without typical spine involvement) | 1.037 | 1.024 | 1.050 | 1.009 | 0.993 | 1.025 | 1.059 | 1.039 | 1.080 | 1.023 | 0.980 | 1.068 | 1.021 | 1.007 | 1.035 | 1.084 | 1.073 | 1.097 | 1.076 | 1.060 | 1.091 | 1.119 | 1.098 | 1.140 | 1.089 | 1.063 | 1.116 | 1.011 | 0.982 | 1.040 |
| Depression | 0.993 | 0.990 | 0.996 | 0.988 | 0.984 | 0.992 | 0.973 | 0.968 | 0.978 | 0.997 | 0.986 | 1.009 | 0.990 | 0.987 | 0.994 | 0.998 | 0.995 | 1.001 | 0.968 | 0.965 | 0.972 | 1.017 | 1.011 | 1.022 | 1.090 | 1.083 | 1.097 | 1.017 | 1.009 | 1.025 |
| Anxiety disorder | 1.007 | 1.002 | 1.013 | 1.020 | 1.014 | 1.027 | 0.974 | 0.966 | 0.982 | 1.032 | 1.013 | 1.051 | 1.041 | 1.035 | 1.046 | 1.052 | 1.047 | 1.057 | 1.044 | 1.037 | 1.050 | 1.081 | 1.072 | 1.089 | 1.072 | 1.062 | 1.083 | 0.967 | 0.955 | 0.979 |
| Psychosomatic disorders | 1.011 | 1.007 | 1.015 | 0.994 | 0.990 | 0.999 | 1.031 | 1.025 | 1.037 | 1.006 | 0.993 | 1.019 | 1.099 | 1.094 | 1.103 | 1.053 | 1.050 | 1.057 | 1.036 | 1.031 | 1.041 | 1.113 | 1.107 | 1.120 | 1.097 | 1.089 | 1.105 | 1.273 | 1.262 | 1.283 |
| Sleep disorders | 1.007 | 1.002 | 1.012 | 0.990 | 0.983 | 0.996 | 1.006 | 0.999 | 1.013 | 1.020 | 1.005 | 1.037 | 0.986 | 0.981 | 0.992 | 1.007 | 1.003 | 1.012 | 0.987 | 0.981 | 0.993 | 1.018 | 1.010 | 1.026 | 1.048 | 1.038 | 1.059 | 0.990 | 0.979 | 1.001 |
| **Diagnoses M40-54** | 1.037 | 1.036 | 1.038 | 0.962 | 0.960 | 0.963 | 1.238 | 1.236 | 1.240 | 1.121 | 1.117 | 1.125 | 1.008 | 1.007 | 1.009 | 1.134 | 1.133 | 1.135 | 1.187 | 1.185 | 1.188 | 1.147 | 1.145 | 1.148 | 1.133 | 1.130 | 1.135 | 1.311 | 1.307 | 1.315 |
| **Physician consultations** |  |  |  |  |  |  |  |  |  |  |  |  |  |  |  |  |  |  |  |  |  |  |  |  |  |  |  |  |  |  |
| General practitioner | 0.934 | 0.931 | 0.937 | 1.018 | 1.015 | 1.021 | 0.870 | 0.866 | 0.874 | 0.974 | 0.964 | 0.985 | 1.084 | 1.081 | 1.087 | 1.073 | 1.071 | 1.076 | 1.042 | 1.039 | 1.046 | 1.067 | 1.063 | 1.072 | 1.258 | 1.250 | 1.265 | 1.097 | 1.089 | 1.105 |
| Orthopedic practitioner | 4.400 | 4.389 | 4.411 | 6.947 | 6.925 | 6.969 | 2.811 | 2.800 | 2.822 | 1.868 | 1.853 | 1.884 | 5.559 | 5.545 | 5.574 | 1.896 | 1.892 | 1.900 | 2.243 | 2.236 | 2.249 | 1.887 | 1.880 | 1.894 | 1.488 | 1.481 | 1.495 | 4.621 | 4.590 | 4.652 |
| Surgeon | 2.159 | 2.151 | 2.167 | 2.548 | 2.537 | 2.558 | 2.038 | 2.027 | 2.050 | 1.455 | 1.436 | 1.475 | 1.362 | 1.356 | 1.369 | 1.382 | 1.376 | 1.387 | 1.511 | 1.503 | 1.518 | 1.403 | 1.393 | 1.413 | 1.170 | 1.159 | 1.182 | 1.317 | 1.303 | 1.332 |
| Preventive and rehabilitation physician | 1.593 | 1.584 | 1.602 | 1.619 | 1.607 | 1.630 | 1.597 | 1.584 | 1.611 | 1.461 | 1.433 | 1.490 | 2.087 | 2.076 | 2.099 | 1.730 | 1.721 | 1.739 | 1.672 | 1.661 | 1.683 | 2.237 | 2.219 | 2.256 | 1.376 | 1.358 | 1.395 | 2.182 | 2.155 | 2.208 |
| **Pain medication** |  |  |  |  |  |  |  |  |  |  |  |  |  |  |  |  |  |  |  |  |  |  |  |  |  |  |  |  |  |  |
| NSAIDs 0.1 to 30 DDD | 1.191 | 1.187 | 1.194 | 1.208 | 1.205 | 1.212 | 1.178 | 1.172 | 1.183 | 1.284 | 1.271 | 1.298 | 1.100 | 1.097 | 1.103 | 1.047 | 1.045 | 1.050 | 1.073 | 1.069 | 1.077 | 1.047 | 1.043 | 1.052 | 1.000 | 0.994 | 1.005 | 1.016 | 1.008 | 1.023 |
| NSAIDs 30 to 90 DDD | 1.258 | 1.254 | 1.262 | 1.205 | 1.201 | 1.210 | 1.413 | 1.407 | 1.420 | 1.559 | 1.543 | 1.575 | 1.100 | 1.097 | 1.104 | 1.120 | 1.117 | 1.123 | 1.164 | 1.160 | 1.169 | 1.110 | 1.105 | 1.116 | 1.079 | 1.072 | 1.086 | 1.053 | 1.045 | 1.062 |
| NSAIDs 90 to 180 DDD | 1.216 | 1.209 | 1.222 | 1.137 | 1.130 | 1.145 | 1.405 | 1.395 | 1.415 | 1.560 | 1.536 | 1.585 | 1.061 | 1.055 | 1.067 | 1.101 | 1.096 | 1.106 | 1.140 | 1.133 | 1.147 | 1.080 | 1.070 | 1.089 | 1.117 | 1.105 | 1.129 | 1.026 | 1.013 | 1.038 |
| NSAIDs 180+ DDD | 1.141 | 1.133 | 1.150 | 1.072 | 1.062 | 1.082 | 1.258 | 1.245 | 1.271 | 1.488 | 1.458 | 1.519 | 0.988 | 0.980 | 0.997 | 1.019 | 1.012 | 1.027 | 1.056 | 1.047 | 1.065 | 0.966 | 0.954 | 0.979 | 1.034 | 1.018 | 1.050 | 0.929 | 0.913 | 0.944 |
| Cox-2 inhibitors 0.1 to 30 DDD | 1.146 | 1.138 | 1.154 | 1.082 | 1.073 | 1.091 | 1.277 | 1.265 | 1.290 | 1.308 | 1.280 | 1.336 | 1.050 | 1.042 | 1.058 | 1.109 | 1.102 | 1.116 | 1.124 | 1.114 | 1.133 | 1.166 | 1.154 | 1.178 | 1.087 | 1.071 | 1.103 | 1.030 | 1.012 | 1.049 |
| Cox-2 inhibitors 30 to 90 DDD | 1.144 | 1.133 | 1.155 | 1.032 | 1.019 | 1.045 | 1.334 | 1.317 | 1.351 | 1.294 | 1.257 | 1.332 | 1.009 | 0.997 | 1.021 | 1.105 | 1.095 | 1.115 | 1.134 | 1.121 | 1.148 | 1.160 | 1.144 | 1.177 | 1.086 | 1.063 | 1.109 | 1.031 | 1.005 | 1.057 |
| Cox-2 inhibitors 90 to 180 DDD | 1.109 | 1.092 | 1.125 | 0.995 | 0.976 | 1.015 | 1.277 | 1.253 | 1.302 | 1.163 | 1.113 | 1.215 | 0.977 | 0.959 | 0.994 | 1.075 | 1.060 | 1.089 | 1.123 | 1.104 | 1.143 | 1.125 | 1.101 | 1.149 | 1.060 | 1.027 | 1.094 | 0.998 | 0.963 | 1.035 |
| Cox-2 inhibitors 180+ DDD | 1.075 | 1.056 | 1.095 | 0.959 | 0.936 | 0.983 | 1.198 | 1.171 | 1.227 | 1.096 | 1.040 | 1.156 | 0.935 | 0.915 | 0.956 | 1.063 | 1.046 | 1.080 | 1.105 | 1.083 | 1.129 | 1.097 | 1.070 | 1.126 | 1.028 | 0.991 | 1.067 | 0.915 | 0.876 | 0.955 |
| Non-opioid analgesics 0.1 to 30 DDD | 1.178 | 1.175 | 1.182 | 1.133 | 1.129 | 1.137 | 1.312 | 1.306 | 1.317 | 1.375 | 1.363 | 1.388 | 1.050 | 1.046 | 1.053 | 1.105 | 1.103 | 1.108 | 1.114 | 1.111 | 1.118 | 1.149 | 1.144 | 1.154 | 1.081 | 1.074 | 1.087 | 1.039 | 1.031 | 1.046 |
| Non-opioid analgesics 30 to 90 DDD | 1.179 | 1.169 | 1.189 | 1.089 | 1.077 | 1.101 | 1.341 | 1.326 | 1.355 | 1.450 | 1.418 | 1.484 | 1.026 | 1.015 | 1.036 | 1.148 | 1.139 | 1.157 | 1.174 | 1.162 | 1.186 | 1.247 | 1.229 | 1.264 | 1.125 | 1.104 | 1.147 | 1.029 | 1.008 | 1.049 |
| Non-opioid analgesics 90 to 180 DDD | 1.130 | 1.106 | 1.155 | 1.050 | 1.019 | 1.082 | 1.245 | 1.210 | 1.280 | 1.317 | 1.245 | 1.393 | 0.993 | 0.966 | 1.021 | 1.173 | 1.149 | 1.197 | 1.209 | 1.179 | 1.239 | 1.315 | 1.270 | 1.362 | 1.070 | 1.019 | 1.125 | 1.045 | 0.996 | 1.097 |
| Non-opioid analgesics 180+ DDD | 1.106 | 1.065 | 1.149 | 1.003 | 0.952 | 1.057 | 1.213 | 1.155 | 1.274 | 1.270 | 1.153 | 1.399 | 1.024 | 0.978 | 1.072 | 1.183 | 1.143 | 1.224 | 1.187 | 1.137 | 1.239 | 1.385 | 1.309 | 1.466 | 1.141 | 1.055 | 1.233 | 0.952 | 0.874 | 1.038 |
| Weak opioids 0.1 to 30 DDD | 1.209 | 1.203 | 1.214 | 1.078 | 1.072 | 1.085 | 1.495 | 1.486 | 1.504 | 1.663 | 1.641 | 1.684 | 1.039 | 1.033 | 1.045 | 1.075 | 1.070 | 1.080 | 1.139 | 1.132 | 1.145 | 1.062 | 1.053 | 1.071 | 0.907 | 0.896 | 0.918 | 1.080 | 1.067 | 1.093 |
| Weak opioids 30 to 90 DDD | 1.186 | 1.175 | 1.197 | 1.014 | 1.001 | 1.027 | 1.463 | 1.446 | 1.480 | 1.612 | 1.574 | 1.650 | 0.970 | 0.958 | 0.982 | 1.043 | 1.033 | 1.054 | 1.109 | 1.096 | 1.122 | 1.034 | 1.016 | 1.052 | 0.896 | 0.874 | 0.918 | 1.050 | 1.026 | 1.075 |
| Weak opioids 90 to 180 DDD | 1.105 | 1.087 | 1.124 | 0.949 | 0.927 | 0.972 | 1.291 | 1.264 | 1.319 | 1.455 | 1.395 | 1.518 | 0.930 | 0.910 | 0.951 | 0.975 | 0.959 | 0.991 | 1.030 | 1.009 | 1.051 | 0.958 | 0.930 | 0.987 | 0.852 | 0.818 | 0.887 | 0.984 | 0.946 | 1.023 |
| Weak opioids 180+ DDD | 1.028 | 1.011 | 1.045 | 0.884 | 0.864 | 0.904 | 1.163 | 1.139 | 1.188 | 1.268 | 1.215 | 1.323 | 0.886 | 0.868 | 0.904 | 0.843 | 0.830 | 0.857 | 0.859 | 0.842 | 0.876 | 0.857 | 0.833 | 0.881 | 0.718 | 0.691 | 0.746 | 0.892 | 0.859 | 0.926 |
| Strong opioids 0.1 to 30 DDD | 1.196 | 1.181 | 1.212 | 0.965 | 0.947 | 0.983 | 1.384 | 1.363 | 1.405 | 1.594 | 1.547 | 1.643 | 0.952 | 0.936 | 0.969 | 1.039 | 1.025 | 1.054 | 1.072 | 1.055 | 1.089 | 1.063 | 1.038 | 1.089 | 0.911 | 0.880 | 0.944 | 1.055 | 1.021 | 1.089 |
| Strong opioids 30 to 90 DDD | 1.148 | 1.120 | 1.176 | 0.945 | 0.911 | 0.979 | 1.237 | 1.201 | 1.274 | 1.534 | 1.450 | 1.622 | 0.915 | 0.885 | 0.947 | 0.989 | 0.965 | 1.014 | 1.018 | 0.988 | 1.050 | 1.042 | 1.000 | 1.086 | 0.876 | 0.824 | 0.932 | 1.032 | 0.974 | 1.093 |
| Strong opioids 90 to 180 DDD | 1.140 | 1.103 | 1.178 | 0.946 | 0.902 | 0.993 | 1.217 | 1.168 | 1.268 | 1.566 | 1.452 | 1.689 | 0.908 | 0.870 | 0.948 | 0.934 | 0.904 | 0.965 | 0.929 | 0.892 | 0.968 | 1.023 | 0.970 | 1.078 | 0.716 | 0.659 | 0.778 | 0.928 | 0.859 | 1.003 |
| Strong opioids 180+ DDD | 1.144 | 1.111 | 1.177 | 0.916 | 0.878 | 0.955 | 1.223 | 1.178 | 1.269 | 1.636 | 1.531 | 1.747 | 0.852 | 0.820 | 0.885 | 0.855 | 0.831 | 0.879 | 0.865 | 0.834 | 0.897 | 0.876 | 0.834 | 0.919 | 0.688 | 0.642 | 0.738 | 0.848 | 0.792 | 0.908 |
| **Sick days leave** |  |  |  |  |  |  |  |  |  |  |  |  |  |  |  |  |  |  |  |  |  |  |  |  |  |  |  |  |  |  |
| No | 1(ref.) |  |  | 1(ref.) |  |  | 1(ref.) |  |  | 1(ref.) |  |  | 1 (ref.) |  |  | 1 (ref.) |  |  | 1 (ref.) |  |  | 1 (ref.) |  |  | 1(ref.) |  |  | 1(ref.) |  |  |
| 1 to 7 days | 1.179 | 1.175 | 1.183 | 1.188 | 1.183 | 1.193 | 1.184 | 1.177 | 1.191 | 1.260 | 1.242 | 1.278 | 1.208 | 1.204 | 1.212 | 1.006 | 1.002 | 1.009 | 1.026 | 1.022 | 1.030 | 1.044 | 1.038 | 1.050 | 0.966 | 0.959 | 0.973 | 0.991 | 0.981 | 1.001 |
| 8 to 21 days | 1.512 | 1.507 | 1.517 | 1.406 | 1.401 | 1.412 | 1.774 | 1.764 | 1.783 | 2.172 | 2.146 | 2.197 | 1.194 | 1.190 | 1.198 | 1.296 | 1.292 | 1.300 | 1.329 | 1.324 | 1.335 | 1.299 | 1.292 | 1.306 | 1.231 | 1.222 | 1.239 | 1.280 | 1.269 | 1.291 |
| 22 to 42 days | 1.806 | 1.799 | 1.814 | 1.477 | 1.469 | 1.484 | 2.725 | 2.709 | 2.741 | 3.644 | 3.598 | 3.690 | 1.194 | 1.188 | 1.200 | 1.544 | 1.538 | 1.550 | 1.669 | 1.661 | 1.678 | 1.491 | 1.480 | 1.501 | 1.377 | 1.364 | 1.390 | 1.528 | 1.512 | 1.544 |
| 42+ days | 1.796 | 1.789 | 1.804 | 1.333 | 1.326 | 1.341 | 3.251 | 3.233 | 3.270 | 4.358 | 4.305 | 4.411 | 1.112 | 1.106 | 1.117 | 1.598 | 1.592 | 1.605 | 1.873 | 1.864 | 1.882 | 1.472 | 1.461 | 1.482 | 1.273 | 1.260 | 1.286 | 1.554 | 1.539 | 1.570 |
| **Education** |  |  |  |  |  |  |  |  |  |  |  |  |  |  |  |  |  |  |  |  |  |  |  |  |  |  |  |  |  |  |
| Unknown school-leaving qualification | 1.004 | 0.999 | 1.010 | 1.046 | 1.035 | 1.057 | 0.966 | 0.958 | 0.975 | 1.089 | 1.065 | 1.114 | 0.959 | 0.954 | 0.964 | 0.951 | 0.946 | 0.955 | 0.947 | 0.941 | 0.953 | 0.903 | 0.896 | 0.911 | 1.059 | 1.047 | 1.071 | 0.925 | 0.912 | 0.938 |
| No school-leaving qualification | 0.987 | 0.979 | 0.996 | 1.017 | 1.007 | 1.028 | 0.906 | 0.893 | 0.919 | 1.064 | 1.031 | 1.099 | 0.930 | 0.921 | 0.939 | 0.875 | 0.867 | 0.883 | 0.865 | 0.855 | 0.875 | 0.785 | 0.772 | 0.799 | 1.045 | 1.025 | 1.065 | 0.919 | 0.899 | 0.939 |
| Lower Secondary leaving certificate | 1.010 | 1.005 | 1.016 | 1.007 | 0.998 | 1.017 | 0.961 | 0.953 | 0.970 | 1.123 | 1.099 | 1.148 | 0.951 | 0.946 | 0.956 | 0.931 | 0.926 | 0.935 | 0.941 | 0.935 | 0.947 | 0.846 | 0.839 | 0.853 | 1.030 | 1.019 | 1.042 | 0.883 | 0.871 | 0.896 |
| Intermediate school leaving certificate | 1.011 | 1.005 | 1.016 | 1.006 | 0.993 | 1.018 | 0.990 | 0.982 | 0.999 | 1.067 | 1.044 | 1.091 | 0.973 | 0.968 | 0.978 | 0.973 | 0.969 | 0.978 | 0.970 | 0.964 | 0.976 | 0.943 | 0.936 | 0.950 | 1.060 | 1.048 | 1.071 | 0.942 | 0.929 | 0.955 |
| High school diploma | 1(ref.) |  |  | 1(ref.) |  |  | 1(ref.) |  |  | 1(ref.) |  |  | 1 (ref.) |  |  | 1 (ref.) |  |  | 1 (ref.) |  |  | 1 (ref.) |  |  | 1(ref.) |  |  | 1(ref.) |  |  |
| Unknown vocational training | 1.019 | 1.010 | 1.028 | 1.017 | 1.011 | 1.024 | 0.960 | 0.944 | 0.970 | 1.074 | 1.034 | 1.114 | 0.964 | 0.956 | 0.972 | 0.902 | 0.895 | 0.908 | 0.895 | 0.886 | 0.905 | 0.882 | 0.871 | 0.892 | 0.999 | 0.982 | 1.016 | 0.962 | 0.941 | 0.984 |
| Without vocational training | 0.984 | 0.976 | 0.993 | 1.016 | 1.005 | 1.027 | 0.902 | 0.890 | 0.915 | 1.039 | 1.001 | 1.078 | 0.958 | 0.950 | 0.966 | 0.888 | 0.882 | 0.895 | 0.880 | 0.872 | 0.889 | 0.821 | 0.811 | 0.831 | 1.033 | 1.015 | 1.051 | 0.932 | 0.912 | 0.953 |
| With vocational training | 1.004 | 0.996 | 1.012 | 1.028 | 1.022 | 1.035 | 0.991 | 0.978 | 1.004 | 1.044 | 1.008 | 1.082 | 0.991 | 0.983 | 0.998 | 1.013 | 1.007 | 1.020 | 1.026 | 1.017 | 1.036 | 1.007 | 0.997 | 1.017 | 1.042 | 1.026 | 1.059 | 0.909 | 0.890 | 0.928 |
| Master craftsman/technician degree | 1.020 | 1.009 | 1.030 | 1.019 | 1.013 | 1.026 | 1.042 | 1.025 | 1.059 | 1.105 | 1.059 | 1.152 | 1.012 | 1.002 | 1.022 | 1.052 | 1.044 | 1.061 | 1.091 | 1.078 | 1.104 | 1.025 | 1.012 | 1.038 | 1.047 | 1.027 | 1.068 | 0.908 | 0.882 | 0.934 |
| College degree | 1(ref.) |  |  | 1(ref.) |  |  | 1(ref.) |  |  | 1(ref.) |  |  | 1 (ref.) |  |  | 1 (ref.) |  |  | 1 (ref.) |  |  | 1 (ref.) |  |  | 1(ref.) |  |  | 1(ref.) |  |  |
| **Occupation** |  |  |  |  |  |  |  |  |  |  |  |  |  |  |  |  |  |  |  |  |  |  |  |  |  |  |  |  |  |  |
| Agriculture | 1(ref.) |  |  | 1(ref.) |  |  | 1(ref.) |  |  | 1(ref.) |  |  | 1 (ref.) |  |  | 1 (ref.) |  |  | 1 (ref.) |  |  | 1 (ref.) |  |  | 1(ref.) |  |  | 1(ref.) |  |  |
| Raw material extraction, production | 1.013 | 1.004 | 1.021 | 1.005 | 0.995 | 1.015 | 1.032 | 1.018 | 1.046 | 1.031 | 1.002 | 1.061 | 1.005 | 0.996 | 1.014 | 0.992 | 0.984 | 0.999 | 0.965 | 0.954 | 0.975 | 0.987 | 0.974 | 0.999 | 1.067 | 1.050 | 1.086 | 1.043 | 1.018 | 1.069 |
| Construction | 1.022 | 1.013 | 1.032 | 1.008 | 0.997 | 1.019 | 1.061 | 1.045 | 1.077 | 1.052 | 1.020 | 1.085 | 1.009 | 1.000 | 1.019 | 0.996 | 0.988 | 1.004 | 0.979 | 0.968 | 0.991 | 0.993 | 0.979 | 1.007 | 1.030 | 1.011 | 1.050 | 1.025 | 0.998 | 1.052 |
| Natural science | 1.002 | 0.990 | 1.014 | 0.977 | 0.963 | 0.992 | 1.055 | 1.035 | 1.076 | 1.033 | 0.990 | 1.078 | 1.022 | 1.009 | 1.035 | 1.017 | 1.006 | 1.029 | 0.997 | 0.982 | 1.012 | 1.052 | 1.032 | 1.072 | 1.002 | 0.977 | 1.028 | 1.018 | 0.984 | 1.053 |
| Transport, logistics and security | 1.016 | 1.007 | 1.025 | 1.011 | 1.001 | 1.022 | 1.028 | 1.014 | 1.042 | 1.042 | 1.012 | 1.072 | 0.986 | 0.978 | 0.995 | 0.955 | 0.947 | 0.962 | 0.922 | 0.912 | 0.932 | 0.953 | 0.940 | 0.965 | 1.033 | 1.016 | 1.051 | 1.046 | 1.021 | 1.072 |
| Commercial services, distribution, tourism | 1.053 | 1.044 | 1.063 | 1.030 | 1.019 | 1.041 | 1.121 | 1.104 | 1.137 | 1.082 | 1.049 | 1.116 | 1.037 | 1.027 | 1.047 | 1.016 | 1.008 | 1.025 | 0.989 | 0.978 | 1.000 | 1.059 | 1.045 | 1.073 | 1.029 | 1.011 | 1.047 | 1.055 | 1.028 | 1.082 |
| Business and law | 1.031 | 1.021 | 1.040 | 0.986 | 0.975 | 0.997 | 1.154 | 1.137 | 1.171 | 1.025 | 0.993 | 1.058 | 1.060 | 1.050 | 1.070 | 1.115 | 1.107 | 1.124 | 1.095 | 1.083 | 1.107 | 1.212 | 1.196 | 1.228 | 1.022 | 1.004 | 1.040 | 0.993 | 0.968 | 1.018 |
| Health, social services, teaching and education | 1.028 | 1.019 | 1.038 | 0.972 | 0.962 | 0.983 | 1.156 | 1.140 | 1.173 | 1.016 | 0.985 | 1.047 | 1.012 | 1.003 | 1.022 | 1.078 | 1.069 | 1.086 | 1.047 | 1.035 | 1.058 | 1.161 | 1.146 | 1.177 | 1.026 | 1.008 | 1.044 | 1.011 | 0.986 | 1.037 |
| Language, media, art, culture and design | 0.996 | 0.982 | 1.009 | 0.968 | 0.952 | 0.983 | 1.057 | 1.034 | 1.080 | 0.950 | 0.901 | 1.000 | 1.054 | 1.040 | 1.068 | 1.030 | 1.018 | 1.042 | 0.996 | 0.980 | 1.012 | 1.115 | 1.094 | 1.136 | 0.968 | 0.943 | 0.995 | 1.057 | 1.018 | 1.097 |
| Military | 1.090 | 0.960 | 1.239 | 1.065 | 0.914 | 1.241 | 0.932 | 0.745 | 1.166 | 1.435 | 0.989 | 2.082 | 1.011 | 0.881 | 1.160 | 0.987 | 0.871 | 1.118 | 0.819 | 0.687 | 0.977 | 1.169 | 0.952 | 1.437 | 1.193 | 0.913 | 1.558 | 1.064 | 0.782 | 1.446 |
| Position „Helper“ | 1(ref.) |  |  | 1(ref.) |  |  | 1(ref.) |  |  | 1(ref.) |  |  | 1 (ref.) |  |  | 1 (ref.) |  |  | 1 (ref.) |  |  | 1 (ref.) |  |  | 1(ref.) |  |  | 1(ref.) |  |  |
| Position „Trained“ | 1.007 | 1.004 | 1.010 | 0.977 | 0.973 | 0.980 | 1.086 | 1.081 | 1.091 | 0.983 | 0.973 | 0.992 | 1.034 | 1.031 | 1.037 | 1.096 | 1.093 | 1.099 | 1.101 | 1.097 | 1.104 | 1.152 | 1.146 | 1.157 | 1.028 | 1.022 | 1.034 | 0.952 | 0.946 | 0.959 |
| Position „Specialist“ | 1.033 | 1.028 | 1.039 | 0.977 | 0.971 | 0.983 | 1.182 | 1.172 | 1.192 | 1.016 | 0.996 | 1.036 | 1.067 | 1.062 | 1.073 | 1.162 | 1.156 | 1.167 | 1.167 | 1.159 | 1.174 | 1.271 | 1.262 | 1.281 | 0.993 | 0.982 | 1.003 | 0.940 | 0.926 | 0.953 |
| Position „Management“ | 1.038 | 1.031 | 1.046 | 0.985 | 0.976 | 0.993 | 1.176 | 1.163 | 1.189 | 1.000 | 0.973 | 1.028 | 1.074 | 1.067 | 1.082 | 1.138 | 1.131 | 1.145 | 1.162 | 1.153 | 1.172 | 1.225 | 1.213 | 1.237 | 0.956 | 0.943 | 0.969 | 0.933 | 0.916 | 0.951 |

Table S6 Poisson regression analysis (bivariate adjusted for 96 spatial planning regions, years and the variable in focus): relative risks for spinal imaging and conservative non-pharmacological spinal therapies from 2012 to 2016 at the patient level. Effects of 96 spatial regions are not shown. Patients with fractures of the spine were excluded.

|  | Diagnostic Imaging | | | X-ray | | | MRI | | | CT | | | SMT (Physician) | | | PT for the spine | | | ET for the spine | | | MT for the spine | | | MA of the spine | | | ACU | | |
| --- | --- | --- | --- | --- | --- | --- | --- | --- | --- | --- | --- | --- | --- | --- | --- | --- | --- | --- | --- | --- | --- | --- | --- | --- | --- | --- | --- | --- | --- | --- |
|  | RR | 95 % CI | | RR | 95 % CI | | RR | 95 % CI | | RR | 95 % CI | | RR | 95 % CI | | RR | 95 % CI | | RR | 95 % CI | | RR | 95 % CI | | RR | 95 % CI | | RR | 95 % CI | |
| ***Sociodemographic*** |  |  |  |  |  |  |  |  |  |  |  |  |  |  |  |  |  |  |  |  |  |  |  |  |  |  |  |  |  |  |
| Female (Ref. Male) | 1.064 | 1.062 | 1.066 | 1.082 | 1.079 | 1.084 | 1.051 | 1.047 | 1.054 | 0.822 | 0.815 | 0.828 | 1.196 | 1.194 | 1.199 | 1.509 | 1.506 | 1.512 | 1.426 | 1.422 | 1.430 | 1.705 | 1.699 | 1.7113 | 1.730 | 1.722 | 1.738 | 1.577 | 1.568 | 1.586 |
| Age group 20-24 | 1(ref.) |  |  | 1(ref.) |  |  | 1(ref.) |  |  | 1(ref.) |  |  | 1 (ref.) |  |  | 1 (ref.) |  |  | 1 (ref.) |  |  | 1 (ref.) |  |  | 1(ref.) |  |  | 1(ref.) |  |  |
| Age group 25-29 | 1.071 | 1.064 | 1.078 | 1.014 | 1.006 | 1.021 | 1.306 | 1.290 | 1.322 | 1.386 | 1.338 | 1.437 | 1.036 | 1.030 | 1.042 | 1.199 | 1.191 | 1.206 | 1.159 | 1.150 | 1.169 | 1.3029 | 1.288 | 1.318 | 1.253 | 1.234 | 1.272 | 1.405 | 1.372 | 1.439 |
| Age group 30-34 | 1.140 | 1.132 | 1.147 | 1.033 | 1.025 | 1.041 | 1.552 | 1.534 | 1.571 | 1.951 | 1.886 | 2.018 | 1.040 | 1.033 | 1.046 | 1.321 | 1.313 | 1.330 | 1.274 | 1.264 | 1.285 | 1.4520 | 1.436 | 1.469 | 1.431 | 1.410 | 1.453 | 1.725 | 1.686 | 1.765 |
| Age group 35-39 | 1.207 | 1.120 | 1.215 | 1.058 | 1.050 | 1.065 | 1.761 | 1.740 | 1.781 | 2.609 | 2.526 | 2.695 | 1.010 | 1.004 | 1.016 | 1.406 | 1.398 | 1.415 | 1.355 | 1.344 | 1.366 | 1.551 | 1.534 | 1.569 | 1.605 | 1.581 | 1.628 | 2.098 | 2.052 | 2.145 |
| Age group 40-44 | 1.246 | 1.239 | 1.254 | 1.064 | 1.057 | 1.072 | 1.900 | 1.879 | 1.922 | 3.163 | 3.066 | 3.263 | 0.983 | 0.977 | 0.988 | 1.490 | 1.481 | 1.499 | 1.444 | 1.433 | 1.455 | 1.634 | 1.617 | 1.652 | 1.764 | 1.740 | 1.789 | 2.451 | 2.399 | 2.503 |
| Age group 45-49 | 1.258 | 1.250 | 1.265 | 1.058 | 1.051 | 1.065 | 1.961 | 1.940 | 1.982 | 3.564 | 3.458 | 3.674 | 0.947 | 0.942 | 0.952 | 1.536 | 1.528 | 1.545 | 1.506 | 1.495 | 1.517 | 1.667 | 1.650 | 1.685 | 1.829 | 1.805 | 1.854 | 2.795 | 2.738 | 2.853 |
| Age group 50-54 | 1.280 | 1.273 | 1.288 | 1.077 | 1.070 | 1.084 | 1.983 | 1.962 | 2.004 | 3.958 | 3.840 | 4.078 | 0.914 | 0.909 | 0.918 | 1.552 | 1.544 | 1.561 | 1.550 | 1.538 | 1.561 | 1.631 | 1.614 | 1.648 | 1.876 | 1.852 | 1.901 | 3.232 | 3.167 | 3.299 |
| Age group 55-59 | 1.290 | 1.283 | 1.298 | 1.094 | 1.087 | 1.101 | 1.946 | 1.925 | 1.967 | 4.325 | 4.197 | 4.457 | 0.869 | 0.864 | 0.874 | 1.541 | 1.532 | 1.549 | 1.558 | 1.547 | 1.570 | 1.550 | 1.534 | 1.567 | 1.907 | 1.881 | 1.933 | 3.785 | 3.708 | 3.863 |
| Age group 60-64 | 1.242 | 1.234 | 1.249 | 1.073 | 1.066 | 1.081 | 1.773 | 1.753 | 1.793 | 4.347 | 4.215 | 4.483 | 0.809 | 0.804 | 0.814 | 1.493 | 1.484 | 1.502 | 1.516 | 1.504 | 1.528 | 1.445 | 1.429 | 1.461 | 1.910 | 1.883 | 1.937 | 4.100 | 4.015 | 4.186 |
| ***Comorbidities*** |  |  |  |  |  |  |  |  |  |  |  |  |  |  |  |  |  |  |  |  |  |  |  |  |  |  |  |  |  |  |
| Osteoarthritis (knee) | 1.199 | 1.194 | 1.204 | 1.146 | 1.140 | 1.152 | 1.320 | 1.311 | 1.329 | 1.495 | 1.474 | 1.517 | 1.151 | 1.146 | 1.156 | 1.218 | 1.213 | 1.223 | 1.293 | 1.286 | 1.299 | 1.132 | 1.125 | 1.140 | 1.327 | 1.316 | 1.339 | 3.982 | 3.953 | 4.011 |
| Osteoarthritis (hip) | 1.499 | 1.490 | 1.507 | 1.471 | 1.461 | 1.481 | 1.692 | 1.678 | 1.706 | 1.907 | 1.874 | 1.940 | 1.317 | 1.309 | 1.325 | 1.324 | 1.317 | 1.330 | 1.484 | 1.475 | 1.494 | 1.229 | 1.218 | 1.240 | 1.240 | 1.226 | 1.255 | 2.461 | 2.432 | 2.490 |
| Osteoporosis | 1.436 | 1.424 | 1.448 | 1.465 | 1.451 | 1.479 | 1.484 | 1.465 | 1.503 | 1.656 | 1.612 | 1.701 | 1.272 | 1.261 | 1.284 | 1.417 | 1.407 | 1.427 | 1.531 | 1.517 | 1.546 | 1.371 | 1.354 | 1.389 | 1.500 | 1.476 | 1.524 | 2.366 | 2.325 | 2.406 |
| Chronic polyarthritis | 1.220 | 1.209 | 1.231 | 1.118 | 1.106 | 1.131 | 1.454 | 1.435 | 1.473 | 1.380 | 1.340 | 1.421 | 1.019 | 1.009 | 1.029 | 1.268 | 1.258 | 1.278 | 1.321 | 1.307 | 1.334 | 1.291 | 1.275 | 1.308 | 1.330 | 1.309 | 1.353 | 1.630 | 1.597 | 1.663 |
| Rheumatic diseases (With typical spine involvement) | 1.235 | 1.222 | 1.247 | 1.103 | 1.089 | 1.117 | 1.657 | 1.634 | 1.681 | 1.173 | 1.130 | 1.217 | 0.934 | 0.923 | 0.945 | 1.302 | 1.290 | 1.313 | 1.499 | 1.483 | 1.516 | 1.267 | 1.248 | 1.287 | 1.038 | 1.016 | 1.061 | 1.326 | 1.292 | 1.360 |
| Rheumatic diseases (Without typical spine involvement) | 1.264 | 1.248 | 1.280 | 1.178 | 1.160 | 1.197 | 1.441 | 1.414 | 1.469 | 1.405 | 1.346 | 1.467 | 1.142 | 1.127 | 1.158 | 1.383 | 1.368 | 1.399 | 1.421 | 1.401 | 1.442 | 1.450 | 1.423 | 1.477 | 1.437 | 1.402 | 1.472 | 1.745 | 1.697 | 1.795 |
| Depression | 1.216 | 1.212 | 1.220 | 1.138 | 1.134 | 1.143 | 1.400 | 1.393 | 1.407 | 1.429 | 1.414 | 1.444 | 1.138 | 1.134 | 1.142 | 1.300 | 1.296 | 1.303 | 1.311 | 1.307 | 1.316 | 1.362 | 1.356 | 1.369 | 1.452 | 1.444 | 1.461 | 1.778 | 1.765 | 1.790 |
| Anxiety disorder | 1.191 | 1.185 | 1.197 | 1.157 | 1.150 | 1.164 | 1.288 | 1.278 | 1.298 | 1.294 | 1.271 | 1.316 | 1.213 | 1.207 | 1.219 | 1.314 | 1.309 | 1.320 | 1.328 | 1.320 | 1.336 | 1.408 | 1.397 | 1.418 | 1.407 | 1.393 | 1.420 | 1.553 | 1.535 | 1.571 |
| Psychosomatic disorders | 1.307 | 1.302 | 1.312 | 1.212 | 1.207 | 1.217 | 1.580 | 1.572 | 1.589 | 1.512 | 1.494 | 1.530 | 1.316 | 1.311 | 1.321 | 1.398 | 1.394 | 1.402 | 1.443 | 1.438 | 1.449 | 1.516 | 1.508 | 1.524 | 1.477 | 1.467 | 1.487 | 2.205 | 2.188 | 2.222 |
| Sleep disorders | 1.210 | 1.204 | 1.216 | 1.117 | 1.111 | 1.124 | 1.401 | 1.391 | 1.411 | 1.550 | 1.527 | 1.574 | 1.067 | 1.061 | 1.072 | 1.207 | 1.201 | 1.212 | 1.240 | 1.233 | 1.247 | 1.214 | 1.205 | 1.223 | 1.291 | 1.279 | 1.304 | 1.661 | 1.643 | 1.679 |
| **Diagnoses M40-54** | 1.183 | 1.182 | 1.184 | 1.109 | 1.108 | 1.110 | 1.438 | 1.436 | 1.440 | 1.400 | 1.395 | 1.404 | 1.104 | 1.103 | 1.105 | 1.233 | 1.232 | 1.234 | 1.306 | 1.305 | 1.307 | 1.242 | 1.240 | 1.244 | 1.241 | 1.237 | 1.243 | 1.613 | 1.609 | 1.616 |
| **Physician consultations** |  |  |  |  |  |  |  |  |  |  |  |  |  |  |  |  |  |  |  |  |  |  |  |  |  |  |  |  |  |  |
| General practitioner | 0.705 | 0.703 | 0.706 | 0.639 | 0.637 | 0.641 | 0.881 | 0.877 | 0.884 | 1.102 | 1.091 | 1.113 | 0.684 | 0.683 | 0.686 | 0.979 | 0.977 | 0.982 | 0.959 | 0.956 | 0.962 | 0.965 | 0.961 | 0.969 | 1.212 | 1.205 | 1.219 | 0.967 | 0.960 | 0.974 |
| Orthopedic practitioner | 5.109 | 5.097 | 5.121 | 7.275 | 7.253 | 7.297 | 4.085 | 4.070 | 4.100 | 2.938 | 2.915 | 2.961 | 5.614 | 5.601 | 5.628 | 2.205 | 2.200 | 2.209 | 2.724 | 2.717 | 2.731 | 2.204 | 2.197 | 2.212 | 1.668 | 1.660 | 1.675 | 5.988 | 5.950 | 6.026 |
| Surgeon | 2.566 | 2.557 | 2.575 | 2.775 | 2.763 | 2.786 | 2.932 | 2.916 | 2.948 | 2.231 | 2.202 | 2.261 | 1.432 | 1.426 | 1.439 | 1.554 | 1.548 | 1.560 | 1.794 | 1.785 | 1.803 | 1.559 | 1.548 | 1.570 | 1.250 | 1.238 | 1.262 | 1.610 | 1.593 | 1.628 |
| Preventive and rehabilitation physician | 2.130 | 2.118 | 2.142 | 2.023 | 2.009 | 2.038 | 2.607 | 2.585 | 2.629 | 2.392 | 2.347 | 2.439 | 2.459 | 2.445 | 2.472 | 2.198 | 2.186 | 2.209 | 2.289 | 2.274 | 2.305 | 2.841 | 2.818 | 2.864 | 1.661 | 1.639 | 1.683 | 3.392 | 3.352 | 3.433 |
| **Pain medication** |  |  |  |  |  |  |  |  |  |  |  |  |  |  |  |  |  |  |  |  |  |  |  |  |  |  |  |  |  |  |
| NSAIDs 0.1 to 30 DDD | 1.468 | 1.464 | 1.472 | 1.522 | 1.517 | 1.526 | 1.466 | 1.460 | 1.473 | 1.623 | 1.606 | 1.639 | 1.301 | 1.298 | 1.305 | 1.120 | 1.118 | 1.123 | 1.182 | 1.178 | 1.186 | 1.106 | 1.102 | 1.111 | 1.040 | 1.034 | 1.045 | 1.217 | 1.209 | 1.226 |
| NSAIDs 30 to 90 DDD | 1.946 | 1.940 | 1.951 | 1.836 | 1.829 | 1.842 | 2.505 | 2.493 | 2.516 | 2.909 | 2.880 | 2.938 | 1.481 | 1.477 | 1.486 | 1.421 | 1.417 | 1.425 | 1.596 | 1.590 | 1.602 | 1.380 | 1.373 | 1.386 | 1.304 | 1.2966 | 1.313 | 1.848 | 1.834 | 1.862 |
| NSAIDs 90 to 180 DDD | 2.110 | 2.100 | 2.120 | 1.842 | 1.831 | 1.854 | 3.158 | 3.136 | 3.180 | 3.781 | 3.726 | 3.838 | 1.473 | 1.465 | 1.481 | 1.571 | 1.564 | 1.578 | 1.820 | 1.809 | 1.830 | 1.501 | 1.489 | 1.514 | 1.514 | 1.498 | 1.529 | 2.406 | 2.378 | 2.434 |
| NSAIDs 180+ DDD | 1.919 | 1.905 | 1.932 | 1.612 | 1.597 | 1.626 | 2.902 | 2.873 | 2.930 | 3.841 | 3.767 | 3.917 | 1.264 | 1.254 | 1.275 | 1.450 | 1.441 | 1.460 | 1.703 | 1.688 | 1.717 | 1.324 | 1.307 | 1.341 | 1.407 | 1.386 | 1.428 | 2.401 | 2.363 | 2.439 |
| Cox-2 inhibitors 0.1 to 30 DDD | 1.726 | 1.715 | 1.738 | 1.581 | 1.568 | 1.595 | 2.237 | 2.216 | 2.259 | 2.315 | 2.267 | 2.364 | 1.392 | 1.382 | 1.403 | 1.451 | 1.442 | 1.461 | 1.594 | 1.581 | 1.608 | 1.507 | 1.492 | 1.523 | 1.337 | 1.317 | 1.357 | 1.746 | 1.715 | 1.778 |
| Cox-2 inhibitors 30 to 90 DDD | 1.905 | 1.887 | 1.924 | 1.594 | 1.574 | 1.614 | 2.838 | 2.802 | 2.874 | 2.859 | 2.779 | 2.942 | 1.385 | 1.370 | 1.402 | 1.593 | 1.578 | 1.607 | 1.822 | 1.800 | 1.844 | 1.654 | 1.623 | 1.677 | 1.442 | 1.412 | 1.473 | 2.153 | 2.100 | 2.207 |
| Cox-2 inhibitors 90 to 180 DDD | 1.877 | 1.850 | 1.905 | 1.499 | 1.470 | 1.528 | 2.957 | 2.902 | 3.013 | 2.798 | 2.680 | 2.921 | 1.313 | 1.290 | 1.337 | 1.627 | 1.605 | 1.649 | 1.915 | 1.882 | 1.949 | 1.698 | 1.662 | 1.734 | 1.4627 | 1.418 | 1.509 | 2.299 | 2.218 | 2.383 |
| Cox-2 inhibitors 180+ DDD | 1.634 | 1.605 | 1.663 | 1.260 | 1.230 | 1.290 | 2.537 | 2.479 | 2.596 | 2.397 | 2.275 | 2.524 | 1.119 | 1.095 | 1.143 | 1.555 | 1.530 | 1.579 | 1.812 | 1.775 | 1.849 | 1.612 | 1.573 | 1.653 | 1.375 | 1.325 | 1.426 | 2.060 | 1.974 | 2.149 |
| Non-opioid analgesics 0.1 to 30 DDD | 1.697 | 1.693 | 1.702 | 1.574 | 1.568 | 1.579 | 2.209 | 2.200 | 2.217 | 2.366 | 2.345 | 2.386 | 1.341 | 1.338 | 1.345 | 1.391 | 1.387 | 1.395 | 1.504 | 1.499 | 1.509 | 1.435 | 1.429 | 1.441 | 1.295 | 1.287 | 1.302 | 1.640 | 1.629 | 1.651 |
| Non-opioid analgesics 30 to 90 DDD | 2.254 | 2.236 | 2.273 | 1.777 | 1.758 | 1.796 | 3.798 | 3.758 | 3.838 | 4.497 | 4.401 | 4.595 | 1.466 | 1.452 | 1.481 | 1.856 | 1.842 | 1.871 | 2.174 | 2.153 | 2.196 | 2.010 | 1.983 | 2.037 | 1.655 | 1.625 | 1.686 | 2.653 | 2.603 | 2.705 |
| Non-opioid analgesics 90 to 180 DDD | 2.089 | 2.044 | 2.135 | 1.548 | 1.503 | 1.595 | 3.643 | 3.544 | 3.744 | 4.392 | 4.158 | 4.639 | 1.310 | 1.274 | 1.346 | 1.875 | 1.837 | 1.913 | 2.227 | 2.173 | 2.283 | 2.116 | 2.044 | 2.190 | 1.542 | 1.468 | 1.620 | 2.836 | 2.705 | 2.974 |
| Non-opioid analgesics 180+ DDD | 1.868 | 1.799 | 1.939 | 1.322 | 1.255 | 1.393 | 3.213 | 3.060 | 3.373 | 3.802 | 3.455 | 4.184 | 1.272 | 1.215 | 1.331 | 1.821 | 1.760 | 1.884 | 2.065 | 1.979 | 2.155 | 2.185 | 2.066 | 2.312 | 1.604 | 1.485 | 1.733 | 2.378 | 2.183 | 2.591 |
| Weak opioids 0.1 to 30 DDD | 2.347 | 2.337 | 2.358 | 1.960 | 1.949 | 1.971 | 3.717 | 3.696 | 3.739 | 4.342 | 4.290 | 4.395 | 1.598 | 1.589 | 1.606 | 1.651 | 1.644 | 1.659 | 1.958 | 1.947 | 1.969 | 1.624 | 1.610 | 1.637 | 1.262 | 1.247 | 1.277 | 2.225 | 2.199 | 2.251 |
| Weak opioids 30 to 90 DDD | 2.398 | 2.376 | 2.420 | 1.764 | 1.742 | 1.786 | 4.346 | 4.297 | 4.395 | 5.257 | 5.139 | 5.377 | 1.419 | 1.402 | 1.436 | 1.732 | 1.715 | 1.748 | 2.125 | 2.100 | 2.149 | 1.700 | 1.671 | 1.728 | 1.343 | 1.312 | 1.376 | 2.588 | 2.529 | 2.648 |
| Weak opioids 90 to 180 DDD | 1.933 | 1.901 | 1.966 | 1.402 | 1.370 | 1.435 | 3.334 | 3.264 | 3.404 | 4.101 | 3.934 | 4.275 | 1.216 | 1.190 | 1.243 | 1.533 | 1.508 | 1.559 | 1.838 | 1.801 | 1.876 | 1.512 | 1.469 | 1.557 | 1.266 | 1.216 | 1.318 | 2.416 | 2.324 | 2.512 |
| Weak opioids 180+ DDD | 1.464 | 1.440 | 1.488 | 1.059 | 1.036 | 1.083 | 2.380 | 2.331 | 2.431 | 2.824 | 2.709 | 2.945 | 0.994 | 0.974 | 1.014 | 1.189 | 1.170 | 1.208 | 1.332 | 1.306 | 1.359 | 1.219 | 1.186 | 1.254 | 1.005 | 0.967 | 1.044 | 1.871 | 1.803 | 1.941 |
| Strong opioids 0.1 to 30 DDD | 2.560 | 2.528 | 2.592 | 1.699 | 1.668 | 1.730 | 4.727 | 4.657 | 4.797 | 6.032 | 5.854 | 6.209 | 1.405 | 1.381 | 1.430 | 1.827 | 1.803 | 1.852 | 2.213 | 2.178 | 2.248 | 1.878 | 1.834 | 1.922 | 1.373 | 1.326 | 1.420 | 2.685 | 2.601 | 2.772 |
| Strong opioids 30 to 90 DDD | 2.203 | 2.150 | 2.257 | 1.432 | 1.382 | 1.484 | 3.930 | 3.817 | 4.047 | 5.414 | 5.122 | 5.718 | 1.216 | 1.176 | 1.257 | 1.711 | 1.669 | 1.754 | 2.046 | 1.985 | 2.108 | 1.853 | 1.779 | 1.932 | 1.357 | 1.277 | 1.443 | 2.723 | 2.572 | 2.883 |
| Strong opioids 90 to 180 DDD | 1.822 | 1.764 | 1.883 | 1.217 | 1.161 | 1.277 | 3.029 | 2.908 | 3.155 | 4.263 | 3.957 | 4.597 | 1.096 | 1.050 | 1.144 | 1.495 | 1.447 | 1.544 | 1.676 | 1.609 | 1.746 | 1.695 | 1.609 | 1.787 | 1.091 | 1.004 | 1.186 | 2.245 | 2.079 | 2.425 |
| Strong opioids 180+ DDD | 1.540 | 1.497 | 1.585 | 0.999 | 0.958 | 1.041 | 2.488 | 2.400 | 2.579 | 3.633 | 3.403 | 3.875 | 0.902 | 0.868 | 0.937 | 1.245 | 1.210 | 1.281 | 1.379 | 1.330 | 1.430 | 1.330 | 1.268 | 1.396 | 0.999 | 0.932 | 1.070 | 1.790 | 1.672 | 1.917 |
| **Sick days leave** |  |  |  |  |  |  |  |  |  |  |  |  |  |  |  |  |  |  |  |  |  |  |  |  |  |  |  |  |  |  |
| No | 1(ref.) |  |  | 1(ref.) |  |  | 1(ref.) |  |  | 1(ref.) |  |  | 1 (ref.) |  |  | 1 (ref.) |  |  | 1 (ref.) |  |  | 1 (ref.) |  |  | 1(ref.) |  |  | 1(ref.) |  |  |
| 1 to 7 days | 1.161 | 1.157 | 1.165 | 1.184 | 1.179 | 1.188 | 1.145 | 1.138 | 1.152 | 1.237 | 1.220 | 1.254 | 1.190 | 1.186 | 1.1934 | 0.923 | 0.920 | 0.926 | 0.944 | 0.940 | 0.948 | 0.947 | 0.942 | 0.953 | 0.872 | 0.866 | 0.878 | 0.845 | 0.837 | 0.854 |
| 8 to 21 days | 1.851 | 1.845 | 1.857 | 1.783 | 1.776 | 1.790 | 2.162 | 2.150 | 2.173 | 2.766 | 2.734 | 2.797 | 1.396 | 1.392 | 1.4011 | 1.338 | 1.334 | 1.342 | 1.438 | 1.432 | 1.444 | 1.291 | 1.285 | 1.298 | 1.255 | 1.246 | 1.263 | 1.534 | 1.521 | 1.547 |
| 22 to 42 days | 2.856 | 2.845 | 2.867 | 2.438 | 2.426 | 2.450 | 4.430 | 4.405 | 4.455 | 6.038 | 5.966 | 6.111 | 1.736 | 1.728 | 1.7446 | 1.871 | 1.863 | 1.878 | 2.210 | 2.199 | 2.221 | 1.740 | 1.728 | 1.753 | 1.598 | 1.584 | 1.613 | 2.566 | 2.540 | 2.592 |
| 42+ days | 3.584 | 3.570 | 3.597 | 2.692 | 2.679 | 2.705 | 7.236 | 7.200 | 7.271 | 9.620 | 9.518 | 9.722 | 1.926 | 1.917 | 1.9351 | 2.304 | 2.296 | 2.313 | 3.053 | 3.039 | 3.067 | 2.098 | 2.084 | 2.113 | 1.696 | 1.680 | 1.712 | 3.676 | 3.643 | 3.710 |
| **Education** |  |  |  |  |  |  |  |  |  |  |  |  |  |  |  |  |  |  |  |  |  |  |  |  |  |  |  |  |  |  |
| Unknown school-leaving qualification | 1.101 | 1.096 | 1.106 | 1.088 | 1.083 | 1.094 | 1.111 | 1.109 | 1.125 | 1.712 | 1.681 | 1.744 | 0.866 | 0.863 | 0.8698 | 0.892 | 0.889 | 0.896 | 0.908 | 0.903 | 0.912 | 0.785 | 0.780 | 0.7900 | 1.140 | 1.130 | 1.151 | 1.188 | 1.174 | 1.201 |
| No school-leaving qualification | 1.130 | 1.121 | 1.140 | 1.127 | 1.116 | 1.138 | 1.135 | 1.097 | 1.126 | 1.980 | 1.924 | 2.038 | 0.824 | 0.817 | 0.8309 | 0.778 | 0.771 | 0.784 | 0.798 | 0.790 | 0.807 | 0.594 | 0.585 | 0.604 | 1.137 | 1.117 | 1.157 | 1.269 | 1.244 | 1.294 |
| Lower Secondary leaving certificate | 1.090 | 1.086 | 1.095 | 1.063 | 1.057 | 1.069 | 1.108 | 1.127 | 1.143 | 1.854 | 1.820 | 1.889 | 0.836 | 0.832 | 0.8397 | 0.873 | 0.870 | 0.877 | 0.921 | 0.916 | 0.926 | 0.714 | 0.710 | 0.719 | 1.074 | 1.064 | 1.084 | 1.085 | 1.072 | 1.098 |
| Intermediate school leaving certificate | 1.062 | 1.057 | 1.066 | 1.041 | 1.035 | 1.047 | 1.117 | 1.010 | 1.116 | 1.397 | 1.370 | 1.424 | 0.923 | 0.919 | 0.9270 | 0.995 | 0.991 | 0.999 | 1.012 | 1.006 | 1.016 | 0.923 | 0.918 | 0.929 | 1.159 | 1.149 | 1.170 | 1.060 | 1.047 | 1.073 |
| High school diploma | 1(ref.) |  |  | 1(ref.) |  |  | 1(ref.) |  |  | 1(ref.) |  |  | 1 (ref.) |  |  | 1 (ref.) |  |  | 1 (ref.) |  |  | 1 (ref.) |  |  | 1(ref.) |  |  | 1(ref.) |  |  |
| Unknown vocational training | 1.102 | 1.094 | 1.110 | 1.120 | 1.110 | 1.129 | 1.060 | 1.049 | 1.072 | 1.725 | 1.673 | 1.778 | 0.847 | 0.841 | 0.8522 | 0.808 | 0.804 | 0.813 | 0.826 | 0.820 | 0.833 | 0.688 | 0.682 | 0.695 | 1.108 | 1.093 | 1.124 | 1.078 | 1.059 | 1.098 |
| Without vocational training | 1.114 | 1.106 | 1.122 | 1.135 | 1.125 | 1.144 | 1.062 | 1.050 | 1.074 | 1.828 | 1.772 | 1.885 | 0.858 | 0.853 | 0.8640 | 0.807 | 0.802 | 0.812 | 0.841 | 0.834 | 0.848 | 0.629 | 0.623 | 0.635 | 1.160 | 1.144 | 1.176 | 1.129 | 1.109 | 1.149 |
| With vocational training | 1.072 | 1.065 | 1.080 | 1.054 | 1.046 | 1.063 | 1.112 | 1.101 | 1.124 | 1.613 | 1.566 | 1.662 | 0.886 | 0.880 | 0.8909 | 0.942 | 0.937 | 0.947 | 0.984 | 0.977 | 0.992 | 0.840 | 0.834 | 0.847 | 1.129 | 1.115 | 1.144 | 0.981 | 0.965 | 0.998 |
| Master craftsman/technician degree | 1.037 | 1.027 | 1.047 | 1.000 | 0.988 | 1.011 | 1.110 | 1.093 | 1.127 | 1.534 | 1.475 | 1.594 | 0.895 | 0.887 | 0.9032 | 0.961 | 0.954 | 0.968 | 0.995 | 0.984 | 1.006 | 0.901 | 0.891 | 0.912 | 1.051 | 1.032 | 1.071 | 0.886 | 0.863 | 0.910 |
| College degree | 1(ref.) |  |  | 1(ref.) |  |  | 1(ref.) |  |  | 1(ref.) |  |  | 1 (ref.) |  |  | 1 (ref.) |  |  | 1 (ref.) |  |  | 1 (ref.) |  |  | 1(ref.) |  |  | 1(ref.) |  |  |
| **Occupation** |  |  |  |  |  |  |  |  |  |  |  |  |  |  |  |  |  |  |  |  |  |  |  |  |  |  |  |  |  |  |
| Agriculture | 1(ref.) |  |  | 1(ref.) |  |  | 1(ref.) |  |  | 1(ref.) |  |  | 1 (ref.) |  |  | 1 (ref.) |  |  | 1 (ref.) |  |  | 1 (ref.) |  |  | 1(ref.) |  |  | 1(ref.) |  |  |
| Raw material extraction, production | 1.039 | 1.030 | 1.048 | 1.035 | 1.024 | 1.045 | 1.058 | 1.044 | 1.073 | 1.024 | 0.995 | 1.054 | 1.038 | 1.029 | 1.048 | 0.994 | 0.987 | 1.002 | 0.988 | 0.977 | 0.999 | 0.970 | 0.958 | 0.983 | 1.055 | 1.037 | 1.073 | 1.092 | 1.066 | 1.118 |
| Construction | 1.032 | 1.022 | 1.041 | 1.009 | 0.998 | 1.020 | 1.093 | 1.077 | 1.109 | 1.091 | 1.058 | 1.125 | 0.996 | 0.986 | 1.005 | 0.914 | 0.906 | 0.922 | 0.938 | 0.927 | 0.949 | 0.871 | 0.858 | 0.883 | 0.863 | 0.847 | 0.879 | 0.933 | 0.909 | 0.958 |
| Natural science | 0.994 | 0.982 | 1.007 | 0.986 | 0.972 | 1.001 | 1.021 | 1.001 | 1.041 | 0.880 | 0.844 | 0.918 | 1.113 | 1.099 | 1.127 | 1.046 | 1.035 | 1.058 | 1.027 | 1.012 | 1.042 | 1.127 | 1.106 | 1.149 | 0.969 | 0.945 | 0.994 | 0.987 | 0.954 | 1.021 |
| Transport, logistics and security | 1.060 | 1.051 | 1.070 | 1.061 | 1.050 | 1.072 | 1.066 | 1.051 | 1.080 | 1.085 | 1.054 | 1.116 | 1.004 | 0.995 | 1.013 | 0.987 | 0.979 | 0.994 | 0.968 | 0.958 | 0.979 | 0.947 | 0.935 | 0.960 | 1.129 | 1.110 | 1.149 | 1.250 | 1.221 | 1.281 |
| Commercial services, distribution, tourism | 1.056 | 1.046 | 1.065 | 1.074 | 1.062 | 1.085 | 1.053 | 1.038 | 1.068 | 0.800 | 0.776 | 0.825 | 1.176 | 1.167 | 1.187 | 1.183 | 1.174 | 1.193 | 1.135 | 1.122 | 1.147 | 1.278 | 1.261 | 1.295 | 1.255 | 1.233 | 1.277 | 1.153 | 1.124 | 1.182 |
| Business and law | 0.982 | 0.974 | 0.991 | 0.976 | 0.966 | 0.986 | 1.021 | 1.007 | 1.036 | 0.680 | 0.659 | 0.701 | 1.202 | 1.191 | 1.214 | 1.339 | 1.328 | 1.349 | 1.278 | 1.264 | 1.292 | 1.555 | 1.535 | 1.575 | 1.260 | 1.238 | 1.282 | 1.095 | 1.068 | 1.123 |
| Health, social services, teaching and education | 1.031 | 1.022 | 1.040 | 1.003 | 0.992 | 1.013 | 1.111 | 1.095 | 1.127 | 0.757 | 0.734 | 0.779 | 1.168 | 1.157 | 1.178 | 1.342 | 1.332 | 1.353 | 1.289 | 1.275 | 1.303 | 1.514 | 1.494 | 1.533 | 1.329 | 1.307 | 1.352 | 1.241 | 1.211 | 1.272 |
| Language, media, art, culture and design | 0.974 | 0.961 | 0.987 | 1.147 | 0.964 | 0.995 | 0.977 | 0.956 | 0.998 | 0.652 | 0.619 | 0.687 | 1.225 | 1.210 | 1.241 | 1.155 | 1.142 | 1.169 | 1.098 | 1.081 | 1.116 | 1.336 | 1.312 | 1.361 | 1.038 | 1.011 | 1.066 | 1.001 | 0.965 | 1.039 |
| Military | 1.166 | 1.027 | 1.325 | 1.147 | 0.984 | 1.337 | 1.001 | 0.800 | 1.252 | 1.316 | 0.908 | 1.908 | 1.172 | 1.021 | 1.344 | 1.103 | 0.973 | 1.249 | 0.953 | 0.799 | 1.137 | 1.297 | 1.056 | 1.593 | 1.240 | 0.950 | 1.620 | 1.251 | 0.920 | 1.700 |
| Position „Helper“ | 1(ref.) |  |  | 1(ref.) |  |  | 1(ref.) |  |  | 1(ref.) |  |  | 1 (ref.) |  |  | 1 (ref.) |  |  | 1 (ref.) |  |  | 1 (ref.) |  |  | 1(ref.) |  |  | 1(ref.) |  |  |
| Position „Trained“ | 0.943 | 0.940 | 0.945 | 0.922 | 0.919 | 0.924 | 0.998 | 0.994 | 1.002 | 0.839 | 0.832 | 0.847 | 1.042 | 1.039 | 1.045 | 1.057 | 1.055 | 1.060 | 1.060 | 1.056 | 1.063 | 1.156 | 1.150 | 1.161 | 0.902 | 0.897 | 0.907 | 0.770 | 0.765 | 0.775 |
| Position „Specialist“ | 0.915 | 0.910 | 0.919 | 0.877 | 0.872 | 0.883 | 1.007 | 0.999 | 1.015 | 0.733 | 0.720 | 0.747 | 1.089 | 1.084 | 1.095 | 1.124 | 1.119 | 1.129 | 1.111 | 1.105 | 1.118 | 1.327 | 1.318 | 1.336 | 0.836 | 0.828 | 0.844 | 0.705 | 0.696 | 0.715 |
| Position „Management“ | 0.908 | 0.902 | 0.914 | 0.877 | 0.871 | 0.884 | 0.979 | 0.969 | 0.988 | 0.642 | 0.626 | 0.658 | 1.133 | 1.126 | 1.140 | 1.150 | 1.144 | 1.156 | 1.140 | 1.132 | 1.149 | 1.371 | 1.359 | 1.382 | 0.819 | 0.809 | 0.829 | 0.752 | 0.740 | 0.765 |

Abbreviations: CT- Computed tomography, MRI- Magnetic resonance imaging, SMT- Spinal manipulative therapy, ACU-acupuncture, PT- Physical therapy, ET-Exercise therapy, MT-Manual therapy, MA-Massage.
